# Characterization of patient-derived site-specific *in vivo* models of pediatric-type diffuse high-grade glioma using magnetic resonance imaging

**DOI:** 10.1093/noajnl/vdag049

**Published:** 2026-02-27

**Authors:** Jessica K R Boult, Diana M Carvalho, Ketty Kessler, Valeria Molinari, Alan Mackay, Yura Grabovska, Mariama Fofana, Kathryn R Taylor, Lynn Bjerke, Elisabet Fernandez, Rita Pereira, Matthew Clarke, Sara Temelso, Anna Burford, Drenusha Sejdiu, Angel M Carcaboso, Julia V Cockle, Fernando Carceller, Lynley V Marshall, Sucheta J Vaidya, Leslie R Bridges, Navneet Singh, Simon Stapleton, Samantha Hettige, Safa Al-Sarraj, Zita Reisz, Bassel Zebian, Cristina Bleil, Richard G Grundy, Juliet C Gray, Darren Hargrave, Shaun Wilson, Susan Picton, Jenny K Adamski, Timothy E G Hassall, Angela Mastronuzzi, Andrea Carai, Philip Benjamin, G Stefania Colafati, Maria Vinci, Chris Jones, Simon P Robinson

**Affiliations:** Centre for Cancer Imaging, Division of Radiotherapy and Imaging, The Institute of Cancer Research, London, UK; Centre for Children and Young People’s Cancer, Division of Cancer Biology, The Institute of Cancer Research, London, UK; Centre for Children and Young People’s Cancer, Division of Cancer Biology, The Institute of Cancer Research, London, UK; Centre for Children and Young People’s Cancer, Division of Cancer Biology, The Institute of Cancer Research, London, UK; Centre for Children and Young People’s Cancer, Division of Cancer Biology, The Institute of Cancer Research, London, UK; Centre for Children and Young People’s Cancer, Division of Cancer Biology, The Institute of Cancer Research, London, UK; Centre for Cancer Imaging, Division of Radiotherapy and Imaging, The Institute of Cancer Research, London, UK; Centre for Children and Young People’s Cancer, Division of Cancer Biology, The Institute of Cancer Research, London, UK; Centre for Children and Young People’s Cancer, Division of Cancer Biology, The Institute of Cancer Research, London, UK; Centre for Children and Young People’s Cancer, Division of Cancer Biology, The Institute of Cancer Research, London, UK; Centre for Children and Young People’s Cancer, Division of Cancer Biology, The Institute of Cancer Research, London, UK; Centre for Children and Young People’s Cancer, Division of Cancer Biology, The Institute of Cancer Research, London, UK; Centre for Children and Young People’s Cancer, Division of Cancer Biology, The Institute of Cancer Research, London, UK; Centre for Children and Young People’s Cancer, Division of Cancer Biology, The Institute of Cancer Research, London, UK; Centre for Children and Young People’s Cancer, Division of Cancer Biology, The Institute of Cancer Research, London, UK; Institut de Recerca Sant Joan de Deu, Barcelona, Spain; Centre for Children and Young People’s Cancer, Division of Cancer Biology, The Institute of Cancer Research, London, UK; Children & Young People’s Unit, The Royal Marsden NHS Foundation Trust, Sutton, UK; Centre for Children and Young People’s Cancer, Division of Cancer Biology, The Institute of Cancer Research, London, UK; Children & Young People’s Unit, The Royal Marsden NHS Foundation Trust, Sutton, UK; Division of Clinical Studies, The Institute of Cancer Research, London, UK; Centre for Children and Young People’s Cancer, Division of Cancer Biology, The Institute of Cancer Research, London, UK; Children & Young People’s Unit, The Royal Marsden NHS Foundation Trust, Sutton, UK; Division of Clinical Studies, The Institute of Cancer Research, London, UK; Centre for Children and Young People’s Cancer, Division of Cancer Biology, The Institute of Cancer Research, London, UK; Children & Young People’s Unit, The Royal Marsden NHS Foundation Trust, Sutton, UK; Division of Clinical Studies, The Institute of Cancer Research, London, UK; Neuropathology, Cellular Pathology, St George’s University Hospitals NHS Foundation Trust, London, UK; Department of Neurosurgery, St George’s University Hospitals NHS Foundation Trust, London, UK; Department of Neurosurgery, St George’s University Hospitals NHS Foundation Trust, London, UK; Department of Neurosurgery, St George’s University Hospitals NHS Foundation Trust, London, UK; Department of Clinical Neuropathology, King’s College Hospital NHS Trust, London, UK (S.A.-S., Z.R.); Department of Clinical Neuropathology, King’s College Hospital NHS Trust, London, UK (S.A.-S., Z.R.); Department of Neurosurgery, King’s College Hospital NHS Foundation Trust, London, UK; Department of Neurosurgery, King’s College Hospital NHS Foundation Trust, London, UK; Children’s Brain Tumour Research Centre, University of Nottingham, Nottingham, UK; Centre for Cancer Immunology, University of Southampton, Southampton, UK; UCL Great Ormond Street Institute for Child Health, London, UK; Department of Haematology and Oncology, Great Ormond Street Hospital for Children NHS Foundation Trust, London, UK; Department of Paediatric Haematology and Oncology, Oxford University Hospitals NHS Foundation Trust, Oxford, UK; Department of Paediatric Oncology and Haematology, Leeds Children’s Hospital, Leeds, UK; Neuro-oncology Division, Birmingham Women’s and Children’s NHS Foundation Trust, Birmingham, UK; Oncology Service, Queensland Children’s Hospital, Brisbane, Australia; Research Area of Onco-haematology and Pharmaceutical GMP Facility, Bambino Gesù Children’s Hospital, IRCCS, Rome, Italy; Neurosurgery Unit, Bambino Gesù Children’s Hospital, IRCCS, Rome, Italy; Department of Neuroradiology, St George’s University Hospitals NHS Foundation Trust, London, UK; Oncological Neuroradiology and Advanced Imaging Unit, Bambino Gesù Children’s Hospital, IRCCS, Rome, Italy; Centre for Children and Young People’s Cancer, Division of Cancer Biology, The Institute of Cancer Research, London, UK; Research Area of Onco-haematology and Pharmaceutical GMP Facility, Bambino Gesù Children’s Hospital, IRCCS, Rome, Italy; Centre for Children and Young People’s Cancer, Division of Cancer Biology, The Institute of Cancer Research, London, UK; Centre for Cancer Imaging, Division of Radiotherapy and Imaging, The Institute of Cancer Research, London, UK

**Keywords:** magnetic resonance imaging, orthotopic xenografts, pediatric-type diffuse high-grade glioma, pre-clinical models

## Abstract

**Background:**

There is an urgent need for novel targeted therapeutic strategies for pediatric-type diffuse high-grade glioma (PDHGG) to improve patient outcomes, the development of which demands model systems that accurately recapitulate the specific PDHGG subtypes. Characterization, longitudinal monitoring and, ultimately, evaluation of treatment response in these models requires sensitive non-invasive imaging techniques such as magnetic resonance imaging (MRI).

**Methods:**

Thirty-five patient-derived, site-specific, orthotopic *in vivo* models of PDHGG, established using implantation of patient tumor material or patient-derived *in vitro* cultures maintained in stem cell retaining conditions, were characterized using multiparametric MRI.

**Results:**

Median survival ranged from 54 to 433 days. Tumors identified on T_2_-weighted (T_2_w) images varied in appearance from a diffuse hyperintense signal to well-defined high contrast masses, and distribution of human nuclear antigen positive tumor cells corresponded to regions of T_2_w signal hyperintensity. Apparent diffusion coefficient was significantly higher in brainstem diffuse midline glioma (DMG) models than in diffuse hemispheric glioma (DHG) tumors, mirroring clinical observations. Lack of contrast-agent enhancement indicated an intact blood-brain barrier in most models, with heterogeneous disruption observed in four DHG models. Upon re-implantation, survival was significantly shortened in 3/4 DHG tumors and 1/10 DMG models, while quantitative MRI parameters remained similar. Furthermore, when 3 models grown in 2D and 3D *in vitro* were implanted in parallel, poorer survival or improved penetrance was associated with 3D cultures.

**Conclusion:**

We established a comprehensive pre-clinical platform in which to evaluate the efficacy of therapeutic strategies against PDHGG *in vivo,* enhanced by the use of multiparametric MRI.

## Graphical Abstract

**Graphical Abstract:**
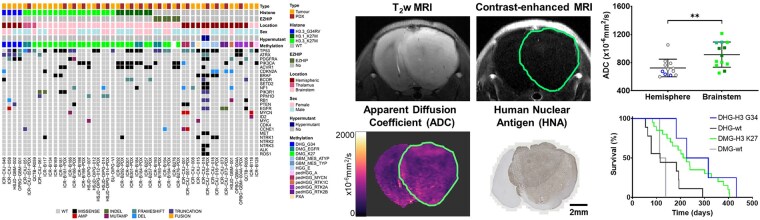

Key Points

- We generated a panel of patient-derived orthotopic *in vivo* models of PDHGG subtypes.
- The growth, survival and MRI phenotype of these *in* vivo PDHGG models is described.
- Our approach provides a robust pre-clinical platform to evaluate therapies for PDHGG.

Importance of Study

Pediatric-type diffuse high-grade gliomas (PDHGG), which have distinct underlying biology to histologically similar tumors occurring in older adults, are a leading cause of tumor-related mortality in children and young adults. There is an urgent need for novel targeted therapeutic strategies to improve patient outcomes, the development of which demands model systems that accurately represent the genotype and phenotype of the specific PDHGG subtypes.

We have established a panel of *in vivo* PDHGG models in mice using site-specific orthotopic injection of patient-derived material from a range of PDHGG subtypes that will provide a more accurate pre-clinical platform in which to evaluate the efficacy of targeted therapeutics. Multiparametric magnetic resonance imaging (MRI) was employed for tumor characterization and longitudinal monitoring; establishing whether the models recapitulated the radiology observed in the clinic, identifying imaging biomarkers that may prove informative in the assessment of treatment response, and reinforcing the importance of imaging-embedded clinical trials.

High-grade glioma, a malignant brain tumor, is a leading cause of tumor-related morbidity and mortality in children and young adults.^1^ In most cases median survival is only 9-18 months, with 2 and 5-year survival rates of 10% and 2%, respectively, in patients with certain subtypes.^2^ The clinical and molecular differences observed in pediatric disease compared with histologically similar lesions in older adults has revealed distinct underlying biology, which differs by anatomical location.^3^^,^^4^ Amongst these are oncohistone H3 mutations,^5^^,^^6^ somatic *ACVR1* mutations,^7–9^ transcriptional dependencies related to the stalled developmental origins of these tumors,^10^^,^^11^ and fusion genes.^12^ The latest WHO classification of central nervous system (CNS) tumors recognizes the distinction of a “pediatric-type” of diffuse high-grade glioma (PDHGG), incorporating several subtypes including diffuse midline glioma, H3 K27-altered (DMG-H3-K27-altered); diffuse hemispheric glioma, H3 G34-mutant (DHG-H3-G34-mutant); infant hemispheric glioma (IHG) driven by single gene fusion events; and a fourth category of PDHGG, H3- and IDH-wildtype, which remains to be fully defined.^13^^,^^14^

There is an urgent need for novel targeted therapeutic strategies for PDHGG to improve patient outcomes, the development of which requires model systems that accurately recapitulate the genotype and phenotype of the specific PDHGG subtypes. Partnerships between clinicians and families, the increasingly widespread ability to biopsy non-resectable tumors within the brainstem,^15^ and more frequent collection of tissue at autopsy,^16^ have increased access to tumor tissue samples for scientists. In addition to improving the understanding of PDHGG disease biology,^3^^,^^4^^,^^17^ such tissue provides opportunities to develop advanced *in vivo* models, utilizing site-specific orthotopic implantation of PDHGG cells, in which to evaluate promising therapeutics. Their systematic use must be underpinned by case-specific evidence for each model, establishing that tumor development, progression and radiology recapitulates the human disease.

Characterization, longitudinal monitoring and, ultimately, evaluation of treatment response in these models requires sensitive non-invasive imaging techniques. Multiparametric magnetic resonance imaging (MRI) is routinely used for diagnosis, surgical/radiotherapy planning, response assessment, and monitoring of pediatric brain tumors. T_1_-weighted (T_1_w) images are routinely acquired pre- and post-administration of a gadolinium-based contrast agent (GBCA) that results in signal enhancement in areas where the blood-brain barrier (BBB) is permeable and the contrast agent can extravasate.^18^^,^^19^ In areas of diffuse infiltrative tumor growth, which can be extensive in PDHGG, the BBB remains intact and does not enhance on T_1_w images. Consequently, there is a higher reliance on T_2_-weighted (T_2_w) and fluid attenuated inversion recovery (FLAIR) images to evaluate tumor extent, which can be challenging due to poorly defined margins. In addition to the guidelines for adult tumors, the inclusion of diffusion-weighted imaging, which can give an indication of hypercellularity for all PDHGG patients, and spinal imaging for patients with DMG in the brainstem (formerly referred to as diffuse intrinsic pontine glioma (DIPG)), is advised.^18^^,^^19^

Here we describe the multiparametric MRI characterization of 35 patient-derived, site-specific, orthotopic *in vivo* models of PDHGG established using either direct implantation of patient tumor material, or implantation of patient-derived *in vitro* cultures maintained in stem cell retaining conditions.

## Methods

### Patient Samples

Patient samples were collected from the South Thames paediatric neurosurgical centres (King’s College Hospital and St George’s Hospital), where the oncology care is delivered at The Royal Marsden Hospital. Fresh tissue of suspected high-grade gliomas from patients aged 1-25 years was obtained at the time of biopsy or resection. Tissues were taken from any anatomical site, and whenever possible, as excess to routine diagnostics. If the pathological diagnosis was not a WHO grade III or IV glioma, the specimen was banked for future appropriate Ethical Committee-approved projects. Samples were collected in Hibernate A transport medium (Thermo Fisher Scientific, Waltham, USA) and processed further in our laboratory. Additional prospectively collected samples were shipped as live minced cryopreserved tissue in Dulbecco’s Modified Eagles Medium/Nutrient Mixture F12 (DMEM/F12; Thermo Fisher Scientific) supplemented with 10% DMSO and 0.1% bovine serum albumin (BSA) or in Hibernate A transport medium at room temperature. Where possible, samples of fresh-frozen tissue and blood were also provided for each case. A summary of samples is provided in Supplementary Table S1.

### Nucleic Acid Extraction

DNA and RNA were extracted following the DNeasy Blood & Tissue kit (QIAGEN, Hilden, Germany) and the RNeasy Plus Mini Kit protocols (QIAGEN), respectively. Occasionally, a dual RNA/DNA extraction kit, Quick-DNA/RNA Miniprep Plus Kit (Zymo Research, Irvine, USA), was used following manufacturer’s instructions. Concentrations were measured using the Qubit dsDNA Assay Kits (Thermo Fisher Scientific) and/or a TapeStation 4200 (Agilent, Santa Clara, USA).

### Methylation Profiling

Methylation analysis was performed using either Illumina 450K or EPIC BeadArrays at University College London (UCL) Great Ormond Street Institute of Child Health, Bart’s Cancer Centre, Bambino Gesu Children’s Hospital, or DKFZ Heidelberg as previously described.^4^^,^^20^ The Heidelberg brain tumor classifier MNP12.8 (https://app.epignostix.com/)^21^ was used to assign a calibrated score to each case, associating it with one of the 185 tumor entities which feature within the current classifier.

Glioma methylation data was assembled from previously published datasets^4^^,^^12^^,^^20^^,^^22^^,^^23^ and publicly available datasets in Array Express (https://www.ebi.ac.uk/biostudies/arrayexpress), GEO (https://www.ncbi.nlm.nih.gov/geo), TCGA (https://portal.gdc.cancer.gov) and Cavatica (https://cavatica.sbgenomics.com/public/projects). 5069 cases with glioma classifications in the MNP12.8 R-package were used as the reference for t-stochastic neighbor embedding (TSNE) based upon the 10K predictor probeset.

### DNA and RNA Sequencing

DNA was sequenced either as whole genome or captured using Agilent SureSelect whole exome v6, xGen Exome Research panel v1 (Integrated DNA Technologies, Leuven, Belgium), or a custom panel of 330 genes known to present in an unselected series of pHGG, as previously described.^4^^,^^20^

Ribosomal RNA depleted from total RNA using NEBNext rRNA Depletion Kit was sequenced as previously described.^4^^,^^20^

### Primary Cell Culture and Tissue Processing

PDHGG patient-derived cultures established at The Institute of Cancer Research or other institutions were grown in stem cell medium as previously described.^20^ Patient-derived cultures were established either immediately after collection or from live cryopreserved tissue,^20^ with authenticity verified using short tandem repeat (STR) DNA fingerprinting (Supplementary Table S2)^24^^,^^25^ and certified mycoplasma-free. Live cryopreserved tissue was processed in a similar manner, with additional cell filtration using 30 µm MACS SmartStrainers (Miltenyi Biotec, Bergisch Gladbach, Germany), prior to direct implantation into mice. Similarly, *in vivo* passages were performed by cryopreserving minced tumor tissue taken from mice in StemCell Banker (AMS Biotechnology Europe, Abingdon, UK) and processing as above for further *in vivo* implantation.

### Orthotopic PDHGG Propagation

All animal experiments were approved by The Institute of Cancer Research Animal Welfare and Ethical Review Body, performed in accordance with the UK Home Office Animals (Scientific Procedures) Act 1986, the United Kingdom National Cancer Research Institute guidelines for the welfare of animals in cancer research,^26^ and reported according to the Animal Research: Reporting *In Vivo* Experiments (ARRIVE) guidelines.^27^ Mice were housed in specific pathogen-free rooms in autoclaved, aseptic microisolator cages with a maximum of 5 animals per cage and were allowed access to food and water *ad libitum*.

Female NOD.Cg-*Prkdc^scid^ Il2rg^tm1WjI^*/SzJ (NSG), NOD.Cg-*Prkdc^scid^*/J (NOD scid) or NCr-*Foxn1^nu^* (athymic nude) mice (Charles River, Harlow, UK) were anesthetized with isoflurane delivered in oxygen (1 %-3%, 1 L/min). Subcutaneous injections of analgesics buprenorphine (0.03 mg/kg, pre-surgery) and meloxicam (5 mg/kg, post-surgery) were given. Animals were depilated, if necessary, at the incision site, and Emla cream 5% (lidocaine/prilocaine) was applied to the skin. The cranium was exposed via midline incision under aseptic conditions, and a 1 mm hole drilled above the injection site using a surgical bone microdrill (Harvard Apparatus, Holliston, USA). Stereotactic apparatus (Harvard Apparatus) was used for site-specific orthotopic implantation at the coordinates: (1) frontal: 1 mm anterior and 2 mm lateral to the bregma at a depth of 2.5 mm from the dura, (2) thalamus: 2 mm posterior and 2 mm lateral to the bregma, depth 3 mm, or (3) pons: 0.8 mm posterior and 1 mm lateral to the lambda, depth 4 mm. 1.5 × 10^5^ to 5 × 10^5^ cells from tumor material or cell cultures were implanted using a 25G 10 µl syringe (VWR, Lutterworth, United Kingdom) at a rate of 2 μl/min using a nanomite syringe pump (Harvard Apparatus). The skin was repaired with tissue adhesive. Mice were monitored until fully recovered from surgery. 24 hour post-surgery a subcutaneous injection of buprenorphine (0.03 mg/kg) was administered.^20^

### MRI Data Acquisition and Analysis

^1^H MRI was performed on a 7T horizontal bore microimaging system (Bruker Biospin, Ettlingen, Germany) using a 30 mm birdcage coil, a 40 mm volume coil or a 20 × 20 mm mouse brain array coil. Anesthesia was induced with 3 % isoflurane in oxygen or medical air (1 L/min) and maintained at 1-2 %. Some animals were anesthetized with a 10 mL/kg intraperitoneal injection of Hypnorm (0.315 mg/mL fentanyl citrate plus 10 mg/ml fluanisone), Hypnovel (5 mg/mL midazolam) and sterile water in a 1:1:2 ratio for endpoint data acquisition. A lateral tail vein was cannulated with a 27G butterfly catheter (Terumo, MidMeds, UK) for remote administration of contrast agent as necessary. Core body temperature was maintained by warm air blown through the magnet bore or using a thermo-regulated water-heated blanket, and breathing rate was monitored using physiological monitoring equipment (SA Instruments, Stony Brook, USA).

Following optimization of the magnetic field homogeneity using a localized map shim over the whole brain, a multi-slice turboRARE T_2_w sequence (repetition time (T_R_) = 4500 ms, effective echo time (T_Eeff_) = 36ms, 1 or 2 averages, RARE factor = 8, in-plane resolution 98 × 98 µm, 1 mm thick contiguous axial, coronal and/or sagittal slices) was used for identification, localisation and longitudinal monitoring of tumors.

When tumors were well established, multiparametric MRI was acquired including; respiratory-gated echo-planar diffusion-weighted imaging (EPI-DWI; T_R_ = 1500 ms, T_E_ = 37.88 ms, 5 b-values 200-1000 s/mm^2^) to determine the apparent diffusion coefficient (ADC),^28^ inversion recovery (IR)-TrueFISP images (T_R_ = 3.4 ms, T_E_ = 1.7 ms) for estimation of native ­relaxation times T_1_ and T_2_, and T_1_-weighted (T_R_ = 1300 ms, T_E_ = 7.5 ms, 4 averages) images acquired before and 2 minutes after intravenous administration of 0.1 mmol/kg gadolinium-based contrast agent (GBCA: Magnevist; Schering, Berlin, Germany, or Dotarem; Guerbet, Villepinte, France) to assess blood-brain barrier integrity. To negate the sensitivity of T_1_ to the level of molecular oxygen dissolved in blood plasma or interstitial tissue fluid, all T_1_/T_2_ mapping was performed using medical air as the carrier for the anesthetic.^29^^,^^30^

Tumor ADC, T_1_ and T_2_ were estimated using a Bayesian maximum *a posteriori* algorithm, which took into account the Rician distribution of noise in magnitude MR data to provide unbiased parameter estimates.^31^^,^^32^ All data were fitted on a pixel-by-pixel basis using in-house software (ImageView, developed in IDL, ITT Visual Information Systems, Boulder, USA), and the median value of each parameter determined from a region of interest (ROI) that encompassed the whole lesion, or the entire imaging slice excluding the ventricles in the case of extremely diffusely growing tumors.

### Immunohistochemistry

Mouse brains were fixed in 10 % neutral buffered formalin for 24 hours, embedded in paraffin, and sectioned (4 μm). Sodium citrate (pH 6.0) heat-mediated antigen retrieval was performed and staining was carried out using antibodies against human nuclear antigen (HNA; clone 3E1.3, Merck, Poole, UK) or Ki67 (clone MIB-1, Agilent, Santa Clara, USA) diluted 1:100 into 1 % Tris buffer with 0.05 % Tween-20 and incubated for 1 hour at room temperature. Novocastra Novolink Polymer Detection Systems Kit (RE7150-CE, Leica Biosystems, Sheffield, UK) was used for detection and slides were mounted using a Leica CV Ultra mounting medium. Slides were imaged using the high throughput-scanning microscope AxioScan Z1 (Zeiss, Oberkochen, Germany) and reviewed by an in-house neuropathologist. ImageJ software and the Cell Counter plugin were used to manually count Ki67-positive and negative cells in 5 randomly selected fields on 3 tumor-containing sections per animal at 400× magnification. The percentage of total cells identified as Ki67 positive for each tissue section were calculated and expressed as the Ki67 proliferation index.

### Statistical Analysis

Statistical and survival analysis was performed using GraphPad Prism 10.1. The mean of median values for quantitative MRI parameters was determined and used for statistical analysis. Results are presented as the mean ± standard deviation (S.D.). Significance testing used Student’s unpaired 2-tailed *t*-test, one-way ANOVA with multiple comparisons, or Fisher’s exact test, survival was evaluated by Log-rank (Mantel-Cox) and relationships between tumor volume and MRI parameters were assessed using simple linear regression. A significance level of 5 % was applied.

## Results

**Figure 1.**
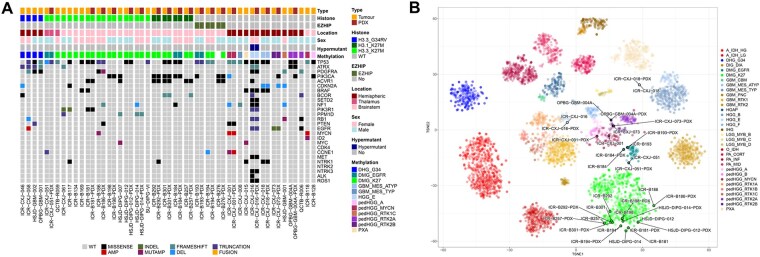
Genomic alterations in patient-derived *in vivo* models of PDHGG. (A) Oncoprint representation of an integrated annotation of single nucleotide variants, DNA copy number changes and structural variants for all originating tumor samples (*n* = 35) and the available *in vivo* models (PDX, *n* = 18). Samples are arranged in columns with genes labelled along rows. Clinicopathological and molecular annotations are provided as bars according to the included key. Histone H3.3 denotes *H3-3A*; H3.1 denotes *H3C2*, *H3C3* or *H3C14*. (B) The *t*-statistic-based stochastic neighbor embedding (t-SNE) projection of a combined methylation data set comprising the matched *in vivo* models (PDX) and tumor samples (circled and labelled). 5069 cases with glioma classifications in the MNP12.8 R-package were used as the reference. The first two projections are plotted on the *x* and *y* axes, with samples represented by dots colored by subtype according to the key provided.

Patient-derived PDHGG cells, taken directly from tumor material or after minimal expansion as stem cell cultures, from 35 samples (34 patients) were implanted into the appropriate anatomical location in immunocompromised mice. The established *in vivo* models were derived from 22 diffuse midline gliomas (DMG), 20 of which were classified as H3-K27-altered (18 from the brainstem and 2 from the thalamus), along with 2 brainstem DMGs that were H3-wt and IDH-wt. H3-K27M mutations were present in *H3-3A* (*H3F3A*; 13 models; 11 brainstem and 2 thalamus), *H3C2* (*HIST1H3B;* 2 models), *H3C3* (*HIST1H3C*) and *H3C14* (*HIST2H3C*) (1 model each) genes, whilst EZHIP overexpression was observed in 3 H3-wt models. Of the 13 models derived from diffuse hemispheric gliomas (DHG), 4 were classified as H3-G34-mutant and 9 as H3-wt and IDH-wt tumors (Figure 1, Table 1, Supplementary Tables S1 and S3).

**Table 1.** Summary of *in vivo* PDHGG models established

Model name	Location	Histone	Model type	DWI	T_1_	T_2_	BBB integrity
**ICR-CXJ-001**	Hemisphere	Wild-type	PCDX-3D; Serial P1+P2	Y	Y	Y	Disrupted
**ICR-CXJ-008**	Hemisphere	Wild-type	PDX	Y	Y	Y	Intact
**ICR-CXJ-015**	Hemisphere	Wild-type	PDX	Y	Y	Y	Intact
**ICR-CXJ-016**	Hemisphere	Wild-type	PDX; Serial P1	Y	Y	Y	Disrupted
**ICR-CXJ-018**	Hemisphere	Wild-type	PDX; Serial P1+P2	Y	Y	Y	Disrupted
**ICR-CXJ-046**	Hemisphere	H3-3A_G34R	PDX	Y	Y	Y	Intact
**ICR-CXJ-051**	Thalamus	H3-3A_K27M	PDX	Y	Y	Y	Intact
**ICR-CXJ-058**	Hemisphere	H3-3A_G34R	PDX	Y	Y	N/A	Intact
**ICR-CXJ-061**	Brainstem	H3-3A_K27M	PDX	Y	Y	Y	Intact
**ICR-CXJ-073**	Hemisphere	Wild-type	PCDX-3D; PDX	Y	Y	Y	Intact
**ICR-B117**	Brainstem	H3-3A_K27M	PDX; Serial P1	N/A	N/A	N/A	N/A
**ICR-B118**	Brainstem	Wild-type	PDX	N/A	N/A	N/A	N/A
**ICR-B128**	Brainstem	Wild-type	PDX	N/A	N/A	N/A	N/A
**ICR-B134**	Brainstem	H3-3A_K27M	PCDX-3D	N/A	N/A	N/A	N/A
**ICR-B169**	Brainstem	H3-3A_K27M	PCDX-3D	Y	N/A	N/A	Intact
**ICR-B181**	Brainstem	H3-3A_K27M	PCDX-3D; PDX; Serial P1	Y	Y	Y	Intact
**ICR-B184**	Brainstem	H3C3_K27M	PCDX-2D; PDX	Y	Y	Y	N/A
**ICR-B186**	Brainstem	H3-3A_K27M	PDX	Y	Y	Y	Intact
**ICR-B193**	Brainstem	EZHIP	PDX; Serial P1	N/A	N/A	N/A	N/A
**ICR-B194**	Brainstem	EZHIP	PDX; Serial P1	Y	Y	Y	N/A
**ICR-B198**	Brainstem	H3-3A_K27M	PDX	N/A	N/A	N/A	N/A
**ICR-B257**	Brainstem	H3C14_K27M	PDX; Serial P1	Y	Y	Y	Intact
**ICR-B276**	Brainstem	EZHIP	PDX; Serial P1	Y	Y	Y	Intact
**ICR-B292**	Brainstem	H3C2_K27M	PDX; Serial P1	Y	Y	Y	Intact
**ICR-B301**	Brainstem	H3C2_K27M	PDX; Serial P1	Y	Y	Y	Intact
**HSJD-DIPG-007**	Brainstem	H3-3A_K27M	PCDX-2D	Y	N/A	N/A	Intact
**HSJD-DIPG-012**	Brainstem	H3-3A_K27M	PCDX-3D; Serial P1+P2	Y	Y	Y	Intact
**HSJD-DIPG-014**	Brainstem	H3-3A_K27M	PCDX-3D; Serial P1+P2	Y	Y	Y	Intact
**HSJD-GBM-001**	Hemisphere	Wild-type	PCDX-2D; PCDX-3D	Y (PCDX-3D)	N/A	N/A	Intact
**HSJD-GBM-002**	Hemisphere	H3-3A_G34R	PCDX-2D; PCDX-3D	Y	N/A	N/A	Intact
**OPBG-GBM-001**	Hemisphere	H3-3A_G34R	PCDX-3D	Y	N/A	N/A	Disrupted (2/3)
**OPBG-GBM-004A**	Hemisphere	Wild-type	PCDX-3D; Serial P1+P2	Y	Y	Y	Intact
**QCTB-R006**	Hemisphere	Wild-type	PCDX-2D	N/A	N/A	N/A	N/A
**QCTB-R059**	Thalamus	H3-3A_K27M	PCDX-2D, PCDX-3D	Y	N/A	N/A	Intact
**SU-DIPG-VI**	Brainstem	H3-3A_K27M	PCDX-2D	N/A	N/A	N/A	Intact

Tumor location, histone subtype, model type and details of the quantitative MRI acquired are shown. More comprehensive details on sample characteristics are presented in Supplementary Table 1. PDX denotes direct implantation of tumor material; PCDX denotes implantation of patient-derived cultured cells (grown in 2D as a monolayer on laminin-coated flasks or in 3D as neurospheres in suspension); Serial P1 denotes mice injected with tumor cells isolated from a mouse from the original (P0) cohort; Serial P2 denotes mice injected with tumor cells from a P1 mouse; DWI = diffusion-weighted imaging; Y = MRI data acquired; N/A = MRI data not acquired.

**Figure 2.**
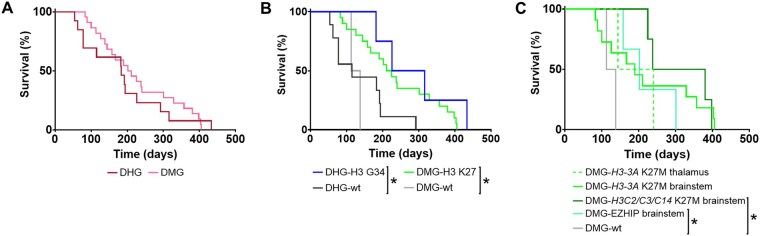
Survival of orthotopic patient-derived PDHGG xenografts. Survival curves for all successfully established orthotopic patient-derived PDHGG models separated by: (A) hemispheric (DHG, *n* = 13, red) or midline (DMG, *n* = 22, pink) location, or (B) location and subtype: DHG-H3-G34-mutant (DHG-H3 G34, *n* = 4, blue); DHG-H3-wt (DHG-wt, *n* = 9, black); DMG-H3-K27-altered (DMG-H3 K27, *n* = 20, green); DMG-H3-wt (DMG-wt, *n* = 2, grey). (C) Survival curves for all DMG models separated by location (thalamus or brainstem) and subtype (*H3-3A* K27M (thalamus *n* = 2, brainstem *n* = 11), *H3C2/H3C3/H3C14* K27M (*n* = 2/1/1, respectively), EZHIP overexpression (*n* = 3), H3-wt (*n* = 2)). Data presented are the median survival for each model using data from first passage (P0) experiments where multiple passages were evaluated, or tumors derived from 3D cultures where both 2D and 3D cultures were implanted and found to be tumorigenic.

Median survival for the individual models ranged from 54 to 433 days from implantation and tumors presented as HNA-positive masses at endpoint (Figure 2, Supplementary Table S1 and Figure S1). Survival analysis, using data from first passage (P0) experiments where multiple passages were evaluated, demonstrated significantly longer median survival in DHG-H3-G34-mutant models than in DHG-H3-wt models (H3-G34-mutant 183 days, H3-wt 115 days, *P* = .039, log-rank (Mantel-Cox); Figure 2B). Longer median survival was also observed in all DMG-H3-K27-altered models compared to DMG-H3-wt models (H3-K27-altered 218 days, H3-wt 125.5 days, *P* = .028; Figure 2B), and in midline DMG-*H3C2/H3C3/H3C14* (H3.1/3.2) K27M and EZHIP overexpressing models when compared to DMG-H3-wt models when stratified by DMG-H3-K27-altered subtypes (H*3C2/H3C3/H3C14* K27M 309 days, EZHIP overexpression 202 days, H3-wt 125.5 days, *P *= .018 and *P* = .039, respectively; Figure 2C).

**Figure 3.**
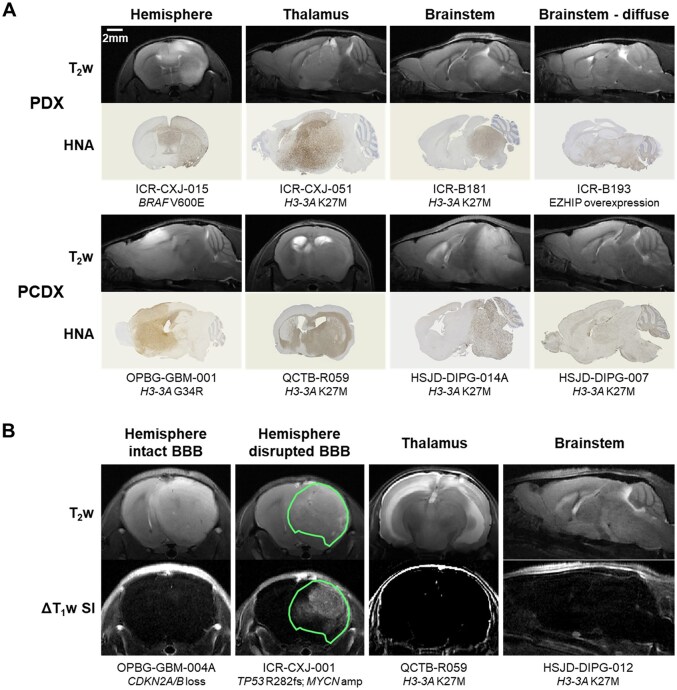
Magnetic resonance imaging and human nuclear antigen immunohistochemistry of patient-derived orthotopic *in vivo* models of PDHGG. (A) T_2_-weighted (T_2_w) MRI and matched human nuclear antigen (HNA) immunohistochemistry of orthotopic patient-derived PDHGG xenografts obtained by site-specific injection of patient tumor tissue directly (PDX) or of PDHGG patient-derived cultured cells (PCDX). Examples of tumors derived from DHG cells injected into the frontal lobe, thalamic DMG cells injected into the thalamus, and brainstem DMG cells injected into the pons, are shown. Scale bar for MRI and histology denotes 2 mm. (B) Maps of the signal intensity change on T_1_-weighted MR images as a result of intravenous injection of gadolinium-based contrast agent (ΔT_1_w SI) alongside matched T_2_w images. Examples of tumors derived from DHG cells injected into the frontal lobe, thalamic DMG cells injected into the thalamus and brainstem DMG cells injected into the pons that lacked tumor signal enhancement, indicative of an intact blood-brain barrier (BBB), are shown. In addition, an example of a DHG tumor demonstrating heterogeneous signal enhancement, indicative of partially disrupted BBB, is shown, with a tumor ROI denoted by a green line.

Tumors identified on T_2_w MR images varied in appearance from a diffuse hyperintense signal to well-defined high contrast masses in models derived both directly from tumor tissue (patient-derived xenograft, PDX) and from stem cell cultures (patient cell line-derived xenograft, PCDX) (Figure 3A, Supplementary Figure S1). HNA staining corresponded to regions of T_2_w signal hyperintensity and tumor cell density correlated with signal intensity where edema was not a factor. The diffuse nature of tumor invasion in 5 of the DMG models (4x H3-K27-altered, 1x H3-wt) and 1 DHG model (H3-wt) resulted in difficulty in tumor identification and delineation using T_2_w MRI and precluded accurate longitudinal monitoring using this technique.

**Figure 4.**
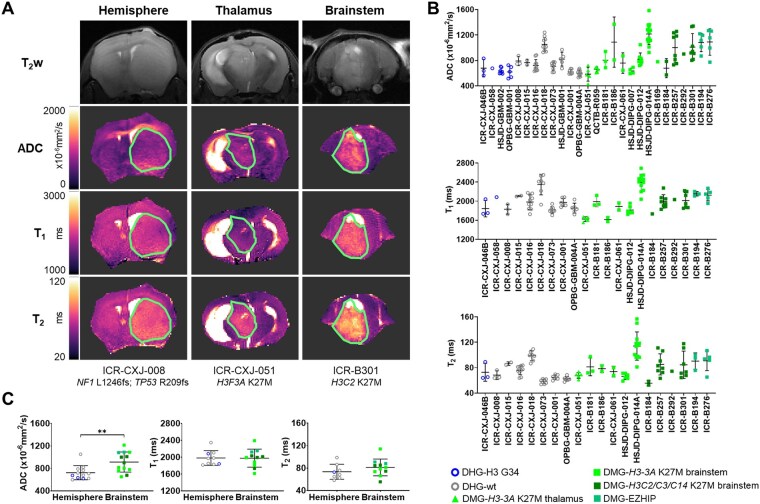
Quantitative multiparametric functional MRI of patient-derived orthotopic *in vivo* models of PDHGG. (A) T_2_-weighted (T_2_w) MR images alongside matched parametric maps of apparent diffusion coefficient (ADC) and relaxation times T_1_ and T_2_ acquired from orthotopic PDHGG xenografts. Tumor ROIs are denoted by a green line. (B) ADC, T_1_, and T_2_ values for all tumors evaluated. Data are mean ± S.D. of individual tumor median values (*n* = 1-15 per model). DHG xenografts are represented by open circles (H3-G34-mutant blue; H3-wt grey); thalamic DMGs by closed triangles (H3-K27-altered green); and brainstem DMGs by closed squares (H3-K27-altered shades of green by subtype). Where serial passages were assessed, all data are included. It was not possible to acquire all parameters from all tumors. (C) Cohort mean ADC, T_1_ and T_2_ values from DHG and brainstem DMG xenografts. Due to small sample numbers, thalamic DMG data (ADC *n* = 2, T_1_/T_2_  *n* = 1) was excluded from these comparisons. Points denote mean of individual tumor median parameter values for >8 models/group (*n* per model 1-15) and plot shows mean ± S.D for each location. Subtype of tumor defined by individual symbols (DHG-H3-G34-mutant blue; DHG-H3-wt grey; DMG-H3-K27-altered shades of green by subtype). Unpaired Student’s t-test, ** *P* < .01.

Where possible, once tumors were well established, mice also underwent multiparametric MRI to further assess their phenotype. Contrast-enhanced T_1_w images following intravenous administration of a GBCA were used to assess the integrity of the BBB. A change in image contrast only occurs if the contrast agent is able to extravasate, indicating a disrupted BBB. No signal enhancement was observed in any of the 14 DMG models imaged using this technique (13x H3-K27-altered, 1x H3-wt) or in 8 DHG (3x H3-G34-mutant, 5x H3-wt) models, consistent with an intact BBB (Figure 3B). Heterogeneous signal enhancement, and therefore BBB disruption in part or all of the tumor mass, was observed in 4 DHG models (1x H3-G34-mutant, 3x H3-wt) (Table 1, Supplementary Table S1). Apparent diffusion coefficient (ADC), derived from diffusion-weighted imaging (DWI) and a measure of water diffusivity, was quantified alongside T_1_ and T_2_ relaxation times from tumor ROIs (Figure 4). Tumor ADC was significantly higher in the 13 brainstem DMG models imaged (11x H3-K27-altered, 2x H3-wt) than in 11 DHG tumors (4x H3-G34-mutant, 7x H3-wt) (*P* = .006, unpaired Student’s *t*-test; Figure 4C). No difference in T_1_ and T_2_ relaxation times were found between the brainstem DMG and DHG models.

Simple linear regression analysis was performed to assess whether the MRI parameters quantified correlated with tumor volume. The MRI values were plotted against ROI size in voxels (156 × 156 × 1000 µm), but as ADC was quantified from three 1 mm thick slices and the T_1_ and T_2_ measurements from one 1 mm thick slice, this did not represent the whole tumor volume in all cases. Assessment of all tumors revealed a weak but significant positive correlation between T_1_ and ROI size (R^2^ = 0.09, *P* = .0036), and in brainstem DMG tumors weak significant correlations were observed between ROI size and T_1_ (R^2^ = 0.15, *P* = .0074) and T_2_ (R^2^ = 0.23, *P* = .0007) (Supplementary Figure S2A). When each model was considered individually, significant positive correlations of assessed tumor volume with ADC were observed in ICR-B276 (R^2^ = 0.89, *P* = .016) and HSJD-DIPG-014A (R^2^ = 0.39, *P* = .04), with T_1_ in ICR-B257 (R^2^ = 0.70, *P* = .0095) and HSJD-DIPG-012 (R^2^ = 0.74, *P* = .027), and with T_2_ in ICR-B257 (R^2^ = 0.81, *P* = .0023) and ICR-B276 (R^2^ = 0.99, *P* = .0008), all of which were brainstem DMG-H3-K27-altered models (Supplementary Figure S2B).

**Figure 5.**
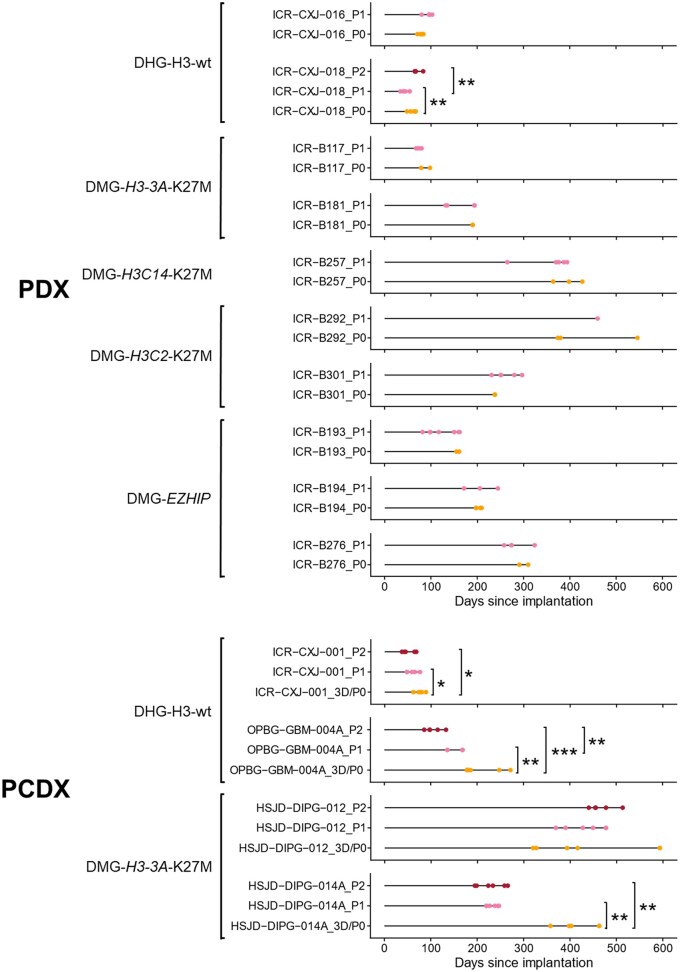
Effect of serial passaging on the survival of PDHGG xenografts. Horizontal plots representing the survival of individual mice bearing serially xenografted PDHGG tumors. Time post implantation is represented on the *x* axis, and each dot represents a single mouse. P0 denotes original cohort of mice injected with cells directly from patient material (orange); 3D/P0 denotes original cohort of mice injected with cells cultured in 3D conditions as neurospheres (orange); P1 denotes mice injected with tumor cells isolated from a P0 mouse (pink); P2 mice were injected with tumor cells from a P1 mouse (red). PDHGG subtype and whether P0 injection was directly from patient tumor tissue (PDX) or from PDHGG patient-derived cultured cells (PCDX) is stated. Log-rank (Mantel-Cox); *P < .05, **P < .01, ***P < .001.

Xenograft tissue from 10 PDX tumors (2x DHG-H3-wt, 8x DMG-H3-K27-altered) and 4 PCDX tumors (2x DHG-H3-wt, 2x DMG-H3-K27-altered) were re-implanted into a further cohort of animals to establish passage 1 (P1) tumors, and for all PCDX-originating tumors and one PDX, P1 tumor tissue was serially implanted to establish P2 tumors. Survival was significantly shortened in three out of four DHG-H3-wt tumors re-implanted (ICR-CXJ-018 (PDX), ICR-CXJ-001 and OPBG-GBM-004A (PCDX)) but only one of the ten DMG-H3-K27-altered models passaged (HSJD-DIPG-014A (PCDX)) (Figure 5, Supplementary Table S1; Log-rank (Mantel-Cox)). No clear changes in T_2_w appearance or contrast-enhancement on T_1_w images were observed between passages in any model. The quantitative MRI parameters assessed remained similar across passages in the majority of models (Supplementary Figure S3), with the only differences being: lower T_1_ in P1 ICR-CXJ-016 DHG-H3-wt tumors compared to P0 PDX tumors (P0: 2124 ± 70 ms, P1: 1945 ± 111 ms, *P* = .045; Student’s *t-*test), and in the ICR-B257 DMG-H3-K27-altered model, lower ADC (P0 (PDX): 1219 ± 48 × 10^−6^mm^2^/s, P1: 871 ± 185 × 10^−6^mm^2^/s, *P* = .045), T_1_ (P0: 2132 ± 125 ms, P1: 1912 ± 61 ms, *P* = .014) and T_2_ (P0: 102.7 ± 10.8 ms, P1: 74.6 ± 7.5 ms, *P* = .045) in the P1 tumors.

**Figure 6.**
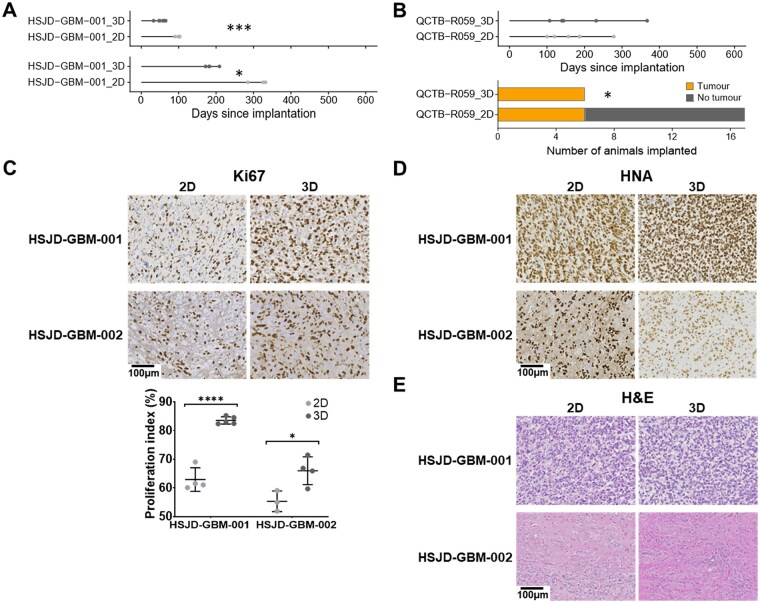
Comparison of survival, take rate and histological characteristics of PDHGG xenografts derived from cells grown in 3D and 2D conditions *in vitro*. (A) Horizontal plots representing the survival of individual mice bearing orthotopic xenografts derived from HSJD-GBM-001 DHG-H3-wt and HSJD-GBM-002 DHG-H3-G34-mutant cells grown in either 3D (dark grey) or 2D (pale grey) conditions *in vitro* with time post implantation represented on the *x* axis. Log-rank (Mantel-Cox); *P < .05, ***P < .001. (B) Horizontal plots representing the survival of individual mice bearing orthotopic xenografts derived from QCTB-R059 thalamic DMG-H3-altered cells grown in either 3D (dark grey) or 2D (pale grey) conditions *in vitro*, presented alongside a representation of the number of mice injected with tumor cells that developed tumors within 1 year of implantation. Fisher’s exact test; *P < .05. (C) Representative images and quantification of immunohistochemical staining for proliferation marker Ki67 from orthotopic xenografts derived from HSJD-GBM-001 and HSJD-GBM-002 DHG cells grown in either 2D or 3D conditions *in vitro*. Data are mean ± S.D. of the percentage Ki67 positive cells in individual tumors. Unpaired Student’s *t*-test, *P < .05, ****P < .0001. Representative images of (D) immunohistochemical staining for human nuclear antigen (HNA), a marker of tumor cells, and (E**)** tinctorial haematoxylin and eosin (H&E) staining of orthotopic xenografts derived from HSJD-GBM-001 and HSJD-GBM-002 DHG cells grown in either 2D or 3D conditions *in vitro*. Ki67 images are not taken from the same tumors as HNA and H&E. Images taken from whole brain images acquired using a 20× objective, scale bar denotes 100 µm.

HSJD-GBM-001 (DHG-H3-wt) and HSJD-GBM-002 (DHG-H3-G34-mutant) cells grown in 2D and 3D conditions *in vitro* were orthotopically implanted in parallel. In both models the median survival of mice bearing tumors derived from cells grown in 3D as neurospheres was markedly and significantly shorter than those with tumors derived from cells grown in 2D as monolayers on laminin (Figure 6A; HSJD-GBM-001: 2D 101 days, 3D 54 days, *P* = .0007; HSJD-GBM-002: 2D 325 days, 3D 182 days, *P* = .02; log-rank (Mantel-Cox)). Quantification of Ki67 staining also demonstrated an increase in the proliferation indices in tumors derived from cells grown in 3D conditions (Figure 6C; HSJD-GBM-001: 2D 62.9 ± 4.1 %, 3D 83.6 ± 1.3 %, *P* < .0001; HSJD-GBM-002: 2D 55.3 ± 3.6 %, 3D 66.0 ± 4.8 %, *P* = .03; Student’s *t-*test). No differences in HNA and H&E staining (Figure 6D and E), or quantitative MRI phenotype were observed between tumors derived from 2D and 3D cultured cells. Whilst there was no difference in survival between mice bearing thalamic DMG-H3-K27-altered QCTB-R059 tumors derived from cells grown in 2D and 3D conditions, there was a significant improvement in tumor take rate when 3D cells were implanted (Figure 6B; 3D 100 %, 2D 35 %, *P* = .014, Fisher’s exact test).

## Discussion

A panel of *in vivo* PDHGG models was established from 35 samples from 34 patients that produced histologically confirmed tumors within 12 or 18 months of site-specific orthotopic implantation in immunocompromised mice (restrictions of the project licenses under which the work was carried out). Of these, 22 were derived directly from dissociated tumor material (PDX) and 16 from tumor cells propagated *in vitro* in stem cell preserving conditions (PCDX); 3 models were successfully propagated as both PDX and PCDX. Genetic and epigenetic analysis of 18 paired tumor and *in vivo* model samples demonstrated that the models represent the original tumors well, maintaining the methylation phenotype and key mutations. The cohort consisted of a higher proportion of DMG samples as many of these originated from the Biological Medicine for DIPG Eradication trial (BIOMEDE; NCT02233049)^33^ for which The Institute of Cancer Research was the UK Translational Research Lead. Only 2 non-brainstem DMGs were propagated, both of which were thalamic DMG-H3-K27-altered, ICR-CXJ-051 and QCTB-R059. DHG-H3-wt models ICR-CXJ-015 and ICR-CXJ-018 were derived from samples taken from the same patient during successive surgical resections 6 months apart. ICR-CXJ-018 tumors, propagated from tissue taken at relapse following radiotherapy and temozolomide treatment, were more rapidly growing and well defined than ICR-CXJ-015 tumors (median survival; ICR-CXJ-018 P0 63 days, ICR-CXJ-015 292 days; *P* = .0018 log-rank (Mantel-Cox)) with significantly higher ADC (ICR-CXJ-018: 1053 ± 109 × 10^−6^mm^2^/s, ICR-CXJ-015: 767 ± 52 × 10^−6^mm^2^/s, *P* = .0054 Student’s *t-*test). Clinical MRI acquired prior to the first surgery is shown alongside imaging of the resulting *in vivo* model in Supplementary Figure S4A. One DHG-H3-wt model, ICR-CXJ-016, was established from a hypermutated tumor from a patient with Constitutional Mismatch Repair Deficiency (CMMRD), whilst this model was amongst the faster growing (median survival 78 days), the histological and imaging parameters assessed were indistinct from other DHG-H3-wt tumors.

In addition to representing a range of molecular subtypes of PDHGG, the *in vivo* models established demonstrated a wide range of growth and MR imaging phenotypes. These ranged from densely cellular expansile growth with minimal invasion into the brain parenchyma, which presented as well defined hyperintense masses on T_2_w MRI and were more commonly observed in DHG models, to diffuse infiltration of the entire brain by tumor cells, which, due to a lesser change in overall cell density were observed as a diffuse slight hyperintensity throughout the brain on T_2_w images, often associated with cerebral expansion. PDX and PCDX tumors arising from ICR-CXJ-073, a DHG-H3-wt tumor resected from the frontal lobe that had spread to the contralateral frontal lobe and leptomeninges at progression (Supplementary Figure S4B), displayed diffuse growth throughout the brain. Expression of pathological variants of EGFR, as detected in this tumor (*EGFR*_N771NPH), has been frequently observed in tumors exhibiting the highly infiltrative gliomatosis cerebri phenotype,^34^ consistent with the invasive growth pattern of the *in vivo* models. All other models that displayed similarly diffuse growth were derived from brainstem DMG tumors, which also commonly present on MRI as extensively diffuse tumors with ill-defined borders (Supplementary Figure S4E).^18^ Additional heterogeneity in tumor growth patterns was observed between DMG models implanted in the brainstem. Some models, such as ICR-B169 and ICR-B194, showed tumor burden restricted primarily to the cerebellum and the pons. Others, for example ICR-B301, showed less tumor in the cerebellum but extended through the brainstem and midbrain, whilst tumor growth of other models, for example ICR-CXJ-061, extended throughout the brain.

Most of the models displayed at least partial diffuse infiltrative growth, and whilst the MRI methods used were informative in most cases, earlier tumor detection, better delineation and quicker screening could be achieved using additional imaging methods. Engineering the tumor cells to express luciferase can facilitate rapid screening and early detection using bioluminescence imaging,^35^ but this requires cells to be cultured *in vitro*, which can change sample heterogeneity, and cells may be altered by the gene insertion. Bioluminescence imaging can also lack spatial information. Metabolic MRI methods are emerging, for example chemical exchange saturation transfer (CEST), that have the potential to improve detection and delineation of diffuse tumor growth.^36^

A wide range of median survival was observed, even within PDHGG molecular subtypes. The DHG models that demonstrated the shortest survival were typically those displaying more expansive, less invasive growth. Mice bearing tumors arising from DHG-H3-G34-mutant samples survived for longer than those with DHG-H3-wt tumors, which mirrors the slightly longer survival observed in patients.^4^ Median overall survival differs between patients with subtypes of DHG-H3-wt, with tumors enriched for *MYCN* amplification exhibiting poorer outcomes than those enriched for *EGFR* or *PDGFRA* amplification.^37^ The only hemispheric pedHGG_MYCN model established demonstrated relatively short median survival (ICR-CXJ-001, 78 days), but HSJD-GBM-001, a *PDGFRA* mutant tumor defined by methylation profiling as pedHGG_RTK1C, was more aggressive (median survival 54 days). Clinical MRI acquired prior to surgery for these patients is shown alongside imaging of the resulting *in vivo* models in Supplementary Figure S4C&F. ICR-B118, a brainstem DMG-H3-wt tumor defined as pedHGG_MYCN, was amongst the DMG models with the shortest median survival (138 days). The slow tumor growth and long survival of the majority of these patient-derived models, even those in which survival was shortened with *in vivo* passaging, are likely to be more representative of clinical disease than more rapidly growing cell line models, but may also present difficulties for therapeutic studies as meaningful results will invariably take longer to achieve.

The majority of the PDHGG models assessed using contrast-enhanced T_1_w MRI presented with an intact BBB across the entire tumor, as evidenced by a lack of GBCA extravasation, and thus indicative of vascular co-option and a lack of neo-angiogenesis.^38^ Clinically, PDHGG tumors often display more heterogeneous BBB integrity,^39^ but, particularly in the case of brainstem DMG, can be almost entirely intact (Supplementary Figure S4).^40^ Diffuse non-enhancing disease is a significant barrier to successful treatment of PDHGG, therefore models that recapitulate this aspect of the tumor phenotype may be beneficial in evaluating therapeutics that can pass through the BBB, and for the assessment of strategies to transiently open the BBB for therapeutic gain.^41^ The small number of DHG xenografts that did exhibit heterogeneous BBB disruption were amongst the fastest growing, cellularly dense and expansile tumors, and while H3-G34-mutant OPBG-GBM-001 tumors were slower growing, this was the most cellularly dense model of this subtype (Clinical MRI shown in Supplementary Figure S4H).

Tumors typically have elevated longitudinal (T_1_) and transverse (T_2_) relaxation times compared with normal tissues, attributed to decreased interactions of intracellular water with proteins and other macromolecules.^42–44^ The presence of edema, in which there is an increase in less structured extracellular water, also contributes to higher T_1_ and T_2_ ­values.^45^^,^^46^ Tumor T_1_ and T_2_ were heterogeneous across the PDHGG models evaluated but were typically higher than the surrounding brain tissue. DWI exploits the Brownian motion of water within tissues, from which tumor ADC, an imaging biomarker often associated with cellularity, is quantified. The inclusion of DWI in standard MRI protocols is recommended for diagnosis, follow-up and response assessment for PDHGG,^18^^,^^19^ but interpretation at a whole tumor level can be complex due to tumor heterogeneity, with relatively unrestricted water diffusion in areas of edema and necrosis present alongside low ADC in areas of high cell density. Tumor ADC was found to be lower in the DHG tumor models than in the brainstem DMG xenografts, which has also been observed between tumors in these locations clinically.^47^ HNA staining showed that DHG xenografts were typically more cellularly dense, consistent with more restricted movement of water, and hence lower ADC. Several studies have associated high ADC metrics in brainstem gliomas at baseline with longer survival, likely due to lower tumor cell density,^48–50^ but efforts to distinguish H3 mutational status in DMG have been inconclusive.^47^^,^^51^ Radiomic approaches, in which multiple imaging parameters are considered together, are likely to show most potential for the discrimination of tumor subtypes,^52^ but these studies are in their relative infancy.

Serial xenografting was achieved in 14 models, with shortened survival observed with passaging in most DHG models but only one DMG model, which was potentially due to the higher tumor cell content in the samples used for reimplantation of DHG tumors. HNA staining showed that the DHG tumors used for passaging were more cellularly dense than the DMG tumors. It is also worth considering that there may be a longer lag time to tumor growth in P0 tumors compared to later passages, particularly PDXs, to allow for more aggressive or tumorigenic clones to be selected for *in vivo* expansion and growth. In most cases the MRI features were similar in tumors from each passage. ADC was lower in P1 ICR-CXJ-016 tumors than P0, and all quantitative MRI parameters were lower in P1 ICR-B257 tumors; in both cases the P1 tumors were smaller at the time of imaging than the P0 tumors, suggesting that tumor size may have influenced these differences rather than a change in tumor phenotype *per se*.

HSJD-GBM-001 and HSJD-GBM-002 DHG xenografts propagated from cells cultured in 3D presented with shorter survival times and higher Ki67 proliferation indices than those established from cells grown in 2D, with no difference in MRI phenotype. Coupled with the higher tumor take rate observed in QCTB-R059 tumors derived from 3D cultures, this suggests that *in vitro* conditions can impact the growth of PDHGG cells *in vivo*. The 3D structures formed when cells are grown in suspension more closely mimic the physical and biochemical microenvironment of a tumor mass; cell-cell interactions, cell morphology and polarity are more likely to be maintained, and stimuli from the local environment will differ from cells grown as monolayers on a laminin matrix.^53^ Therefore, cells grown in 3D may be better primed for growth *in vivo* than those grown in 2D.

In conclusion, this study describes the growth, survival and MR imaging characteristics of a large panel of patient-derived orthotopic *in vivo* models of a range of PDHGG subtypes. The models presented provide a more accurate pre-clinical platform in which to evaluate the efficacy of urgently needed novel therapeutics for the treatment of PDHGG *in vivo*, and which can be assessed using multiparametric MRI.

## Supplementary Material

vdag049_Supplementary_Data

## Data Availability

Sequencing data have been deposited in the European Genome–phenome Archive (www.ebi.ac.uk/ega), and methylation array data have been deposited in ArrayExpress (www.ebi.ac.uk/arrayexpress) with accession numbers E-MTAB-16762 (EPIC) and E-MTAB-16763 (450K). Models may be requested, subject to available stocks, from the originating laboratories. For ICR- and QCTB- cells, which are available under standard MTA, please contact Chris Jones (chris.jones@icr.ac.uk); For SU- cells, please contact Michelle Monje (mmonje@stanford.edu); for HSJD- cells, please contact Angel Montero Carcaboso (amontero@fsjd.org); for OPBG- cells, please contact Maria Vinci (maria.vinci@opbg.net). Imaging and histological data are available upon request from the corresponding author.
