# Supplementary material for: Characterization of patient-derived site-specific in vivo models of pediatric-type diffuse high-grade glioma using magnetic resonance imaging
Source: Neurooncol Adv. 2026 Feb 27;8(1):vdag049. doi: 10.1093/noajnl/vdag049 (PMC13023044; doi:10.1093/noajnl/vdag049)
Supplement: vdag049_Supplementary_Data [file vdag049_supplementary_data.zip › Boult et al_supplementary_revision_clean.docx]

**Supplementary Tables and Figures**

**Supplementary Table S1:** List of all patient-derived PDHGG models implanted *in vivo* with basic phenotypic and molecular annotation, details of *in vivo* tumourigenicity and survival, and the multiparametric magnetic resonance imaging (MRI) acquired.

**Supplementary Table S2:** Short tandem repeat (STR) DNA fingerprinting profiles for all patient-derived PDHGG models implanted *in vivo.*

**Supplementary Table S3:** Data table for Figure 1 listing sample characteristics, methylation classification scores and genetic alterations represented in the oncoprint.

**Supplementary Figure S1**

**
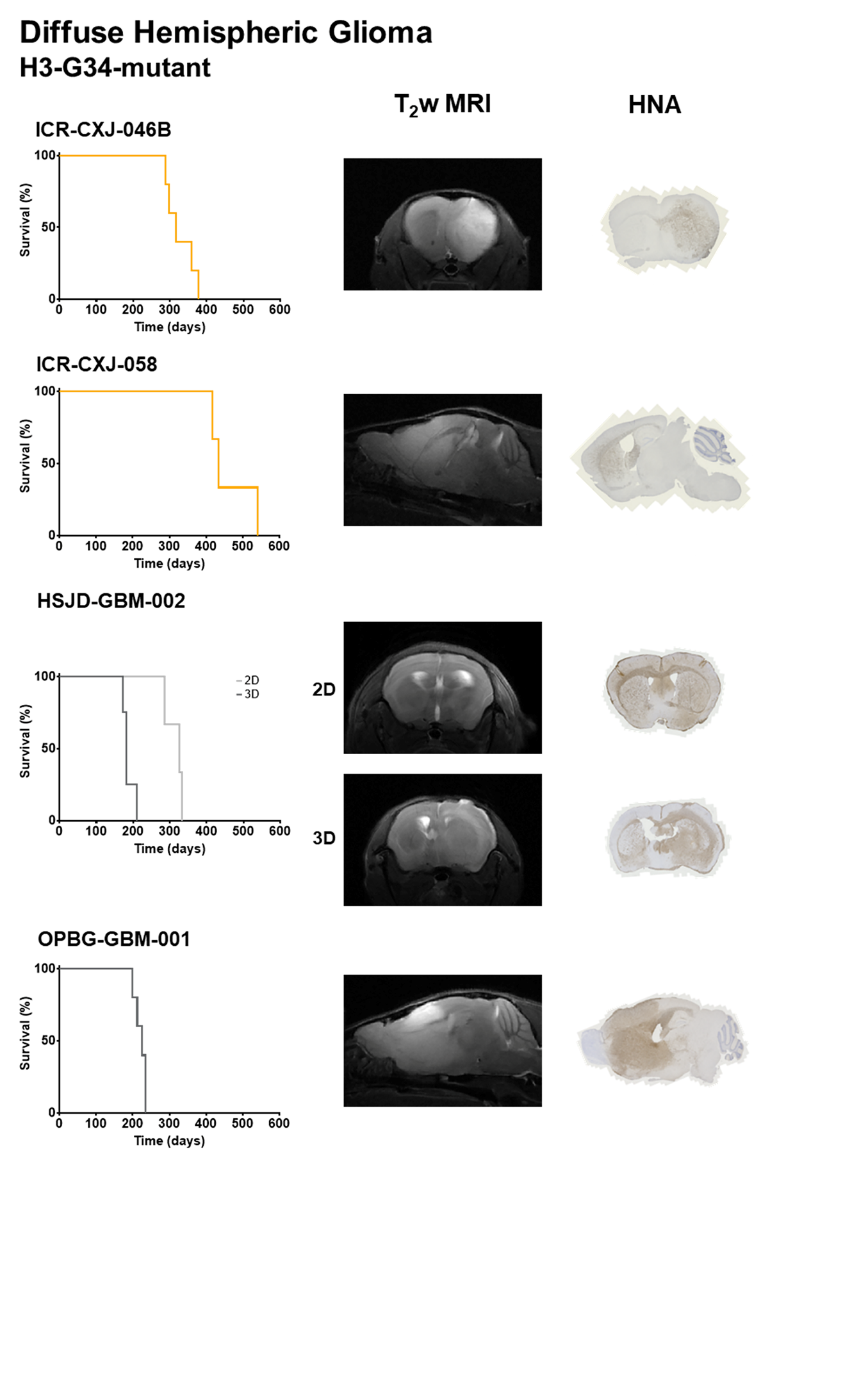
**

**Supplementary Figure S1**

*
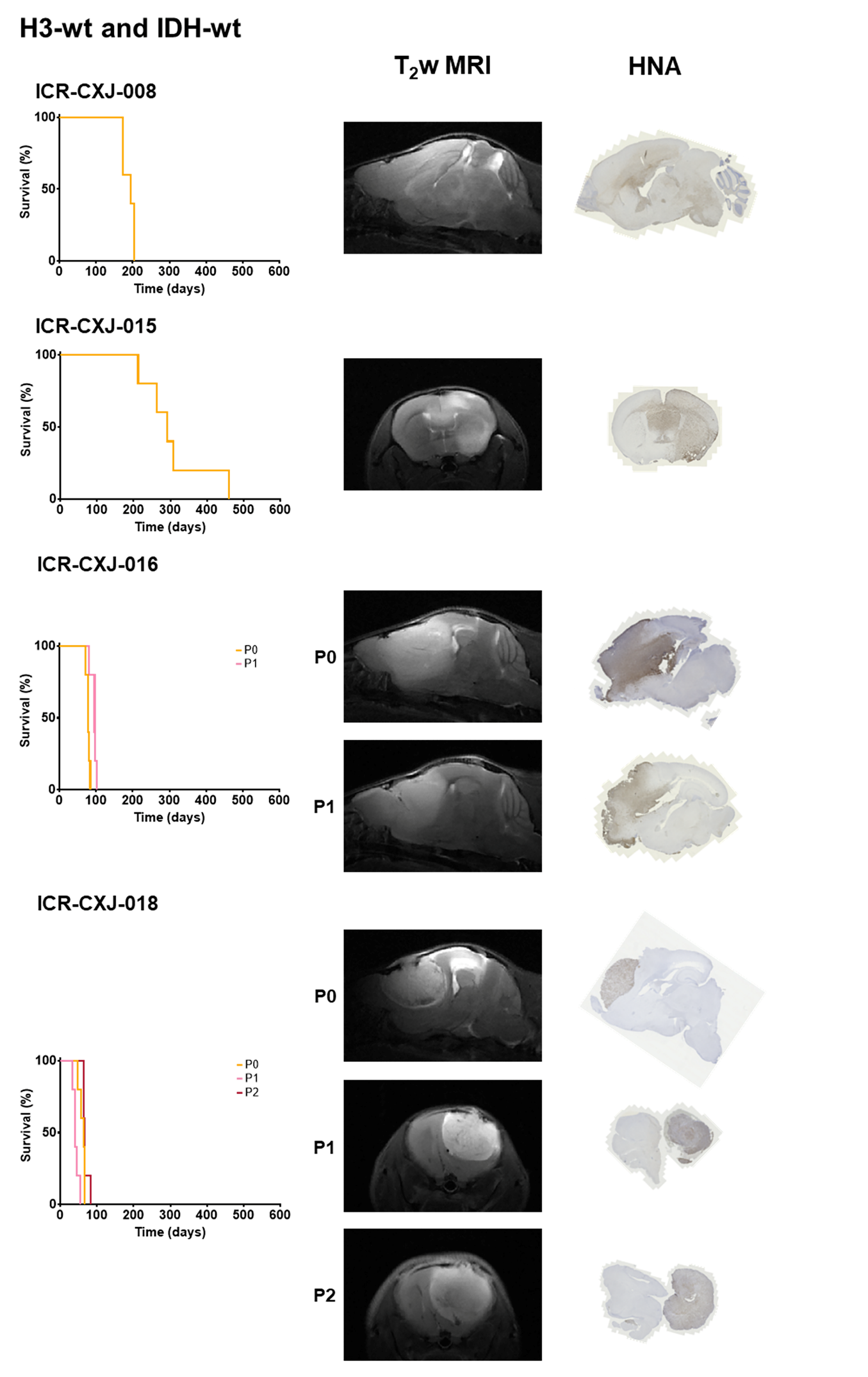
*

**Supplementary Figure S1**

*
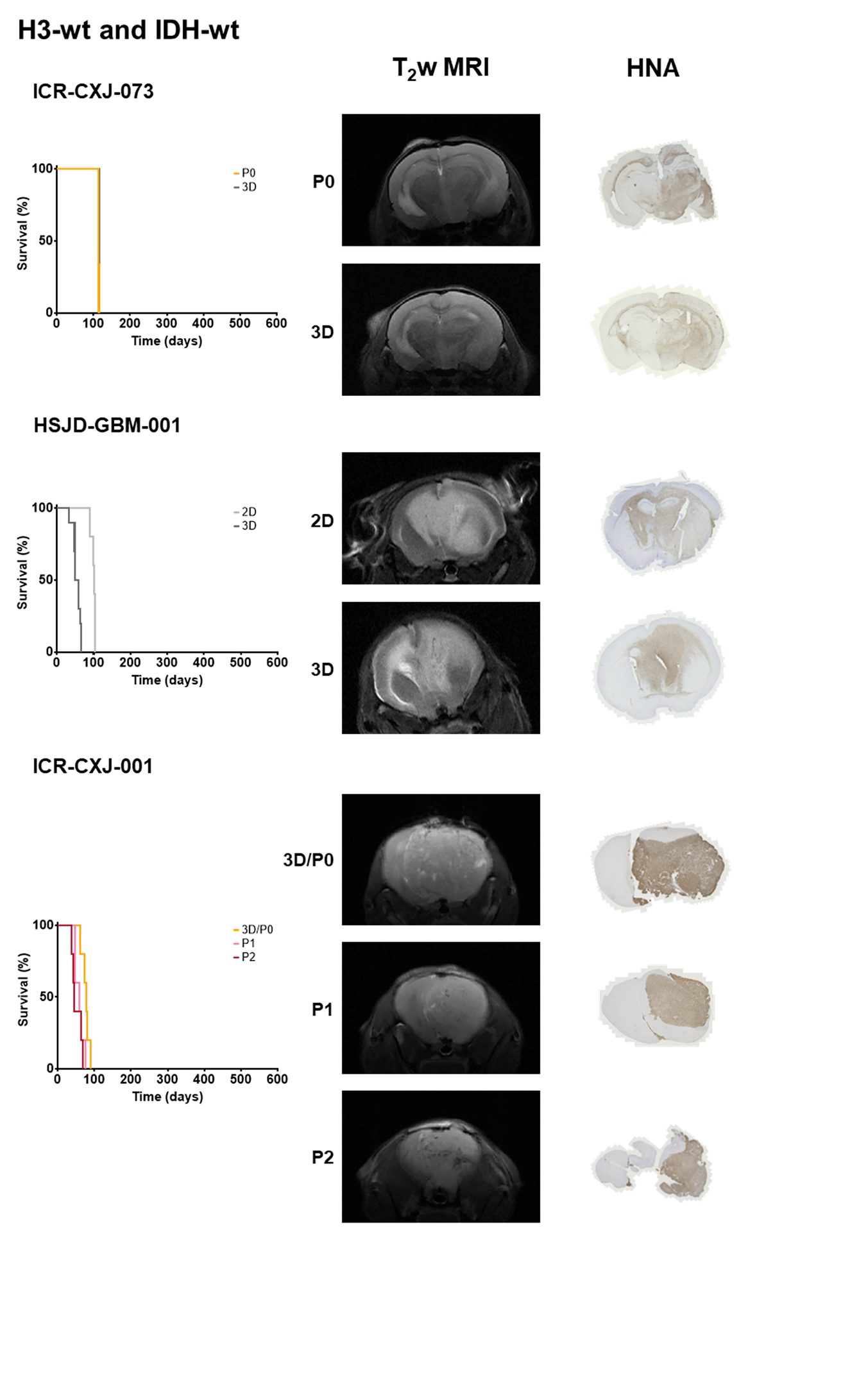
*

**Supplementary Figure S1**

*
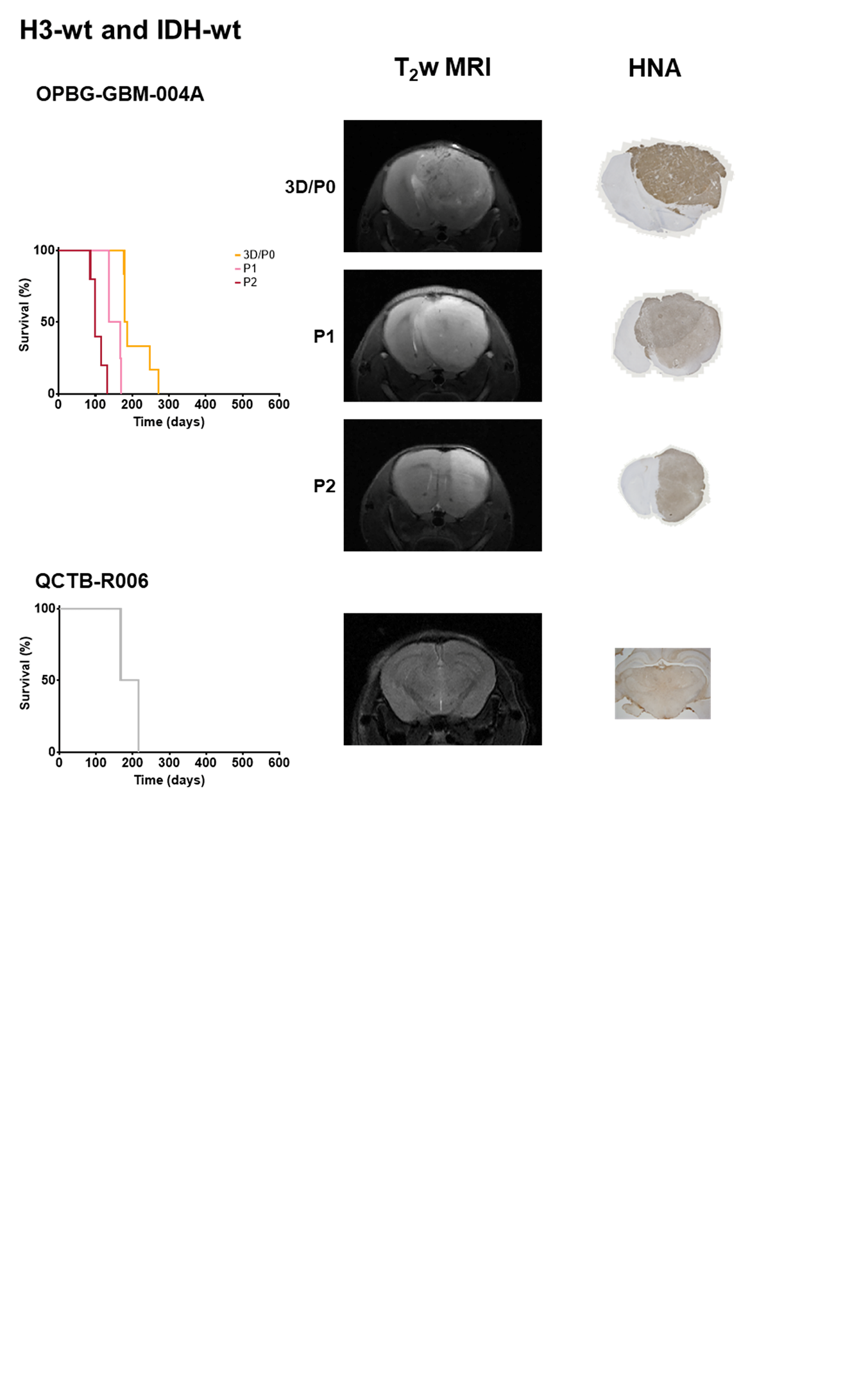
*

**Supplementary Figure S1**

*
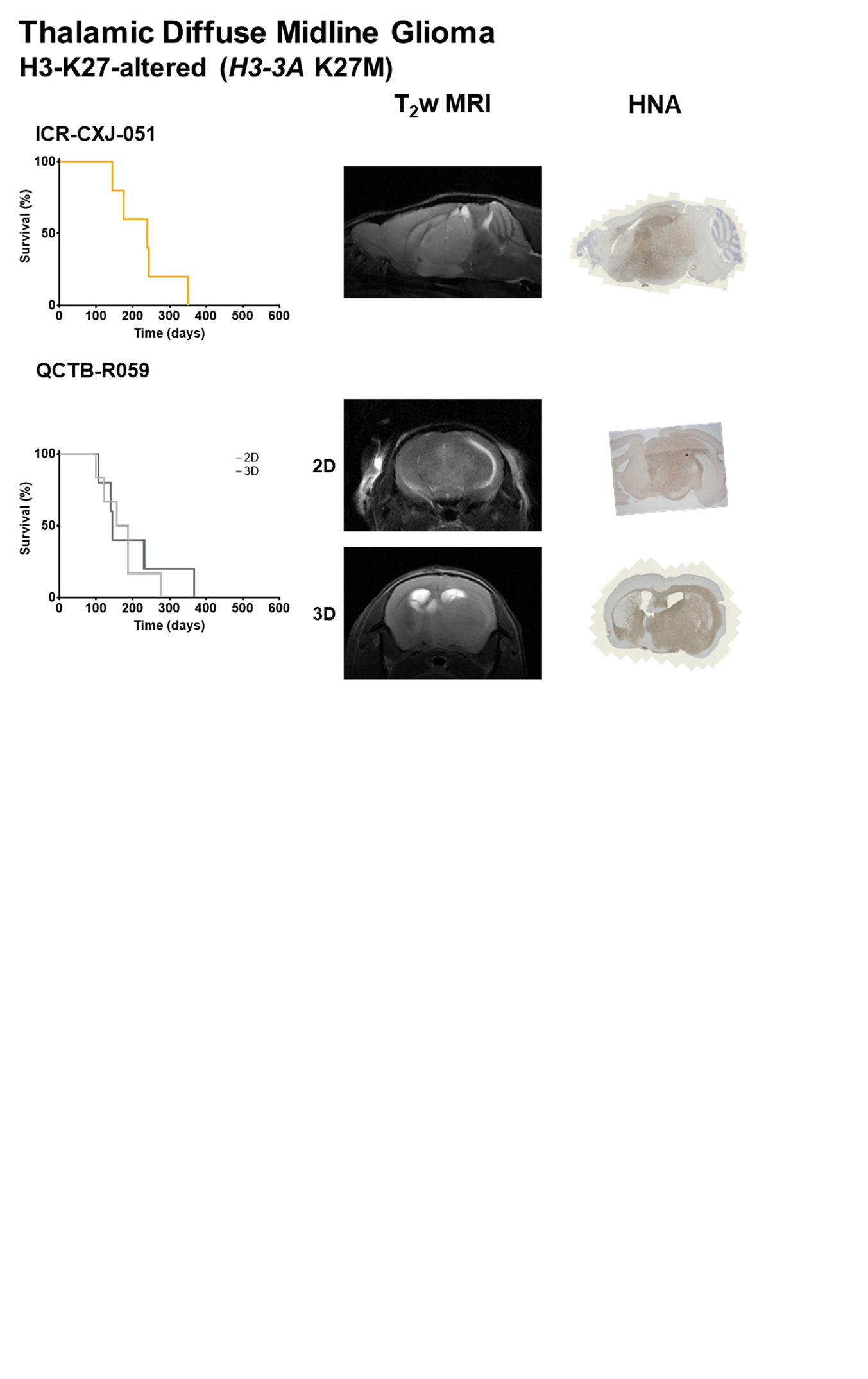
*

**Supplementary Figure S1**

*
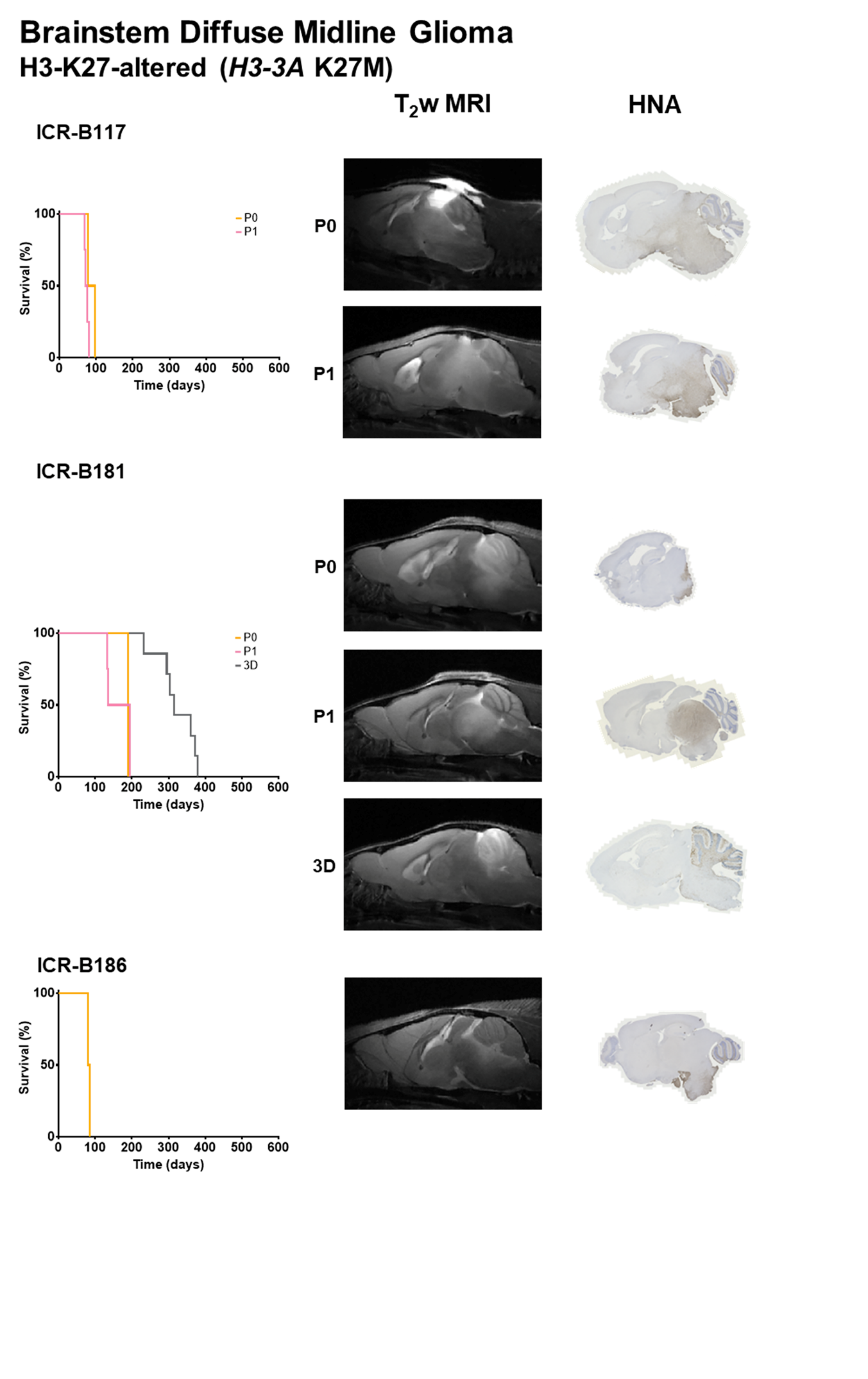
*

**Supplementary Figure S1**

*
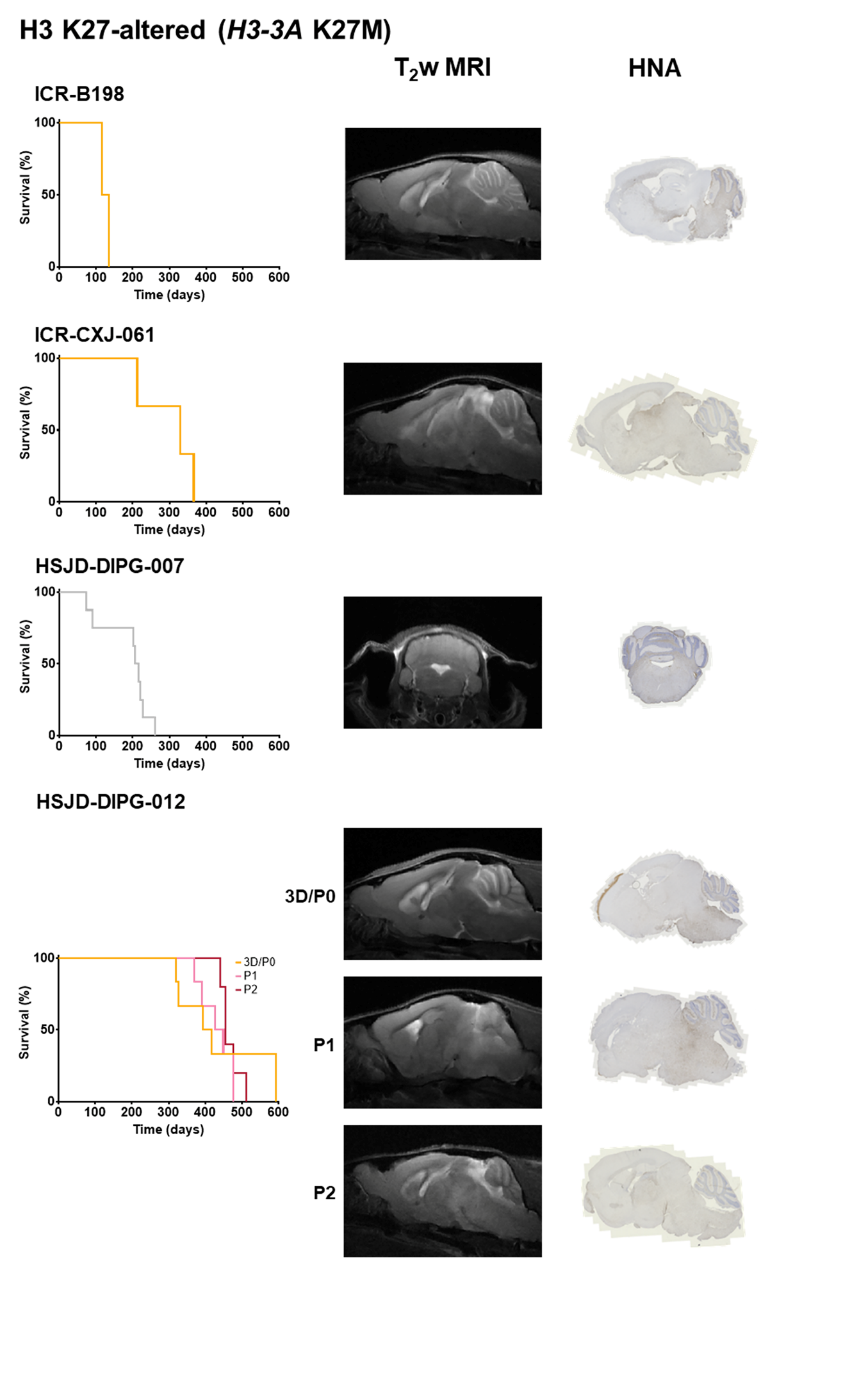
*

**Supplementary Figure S1**

*
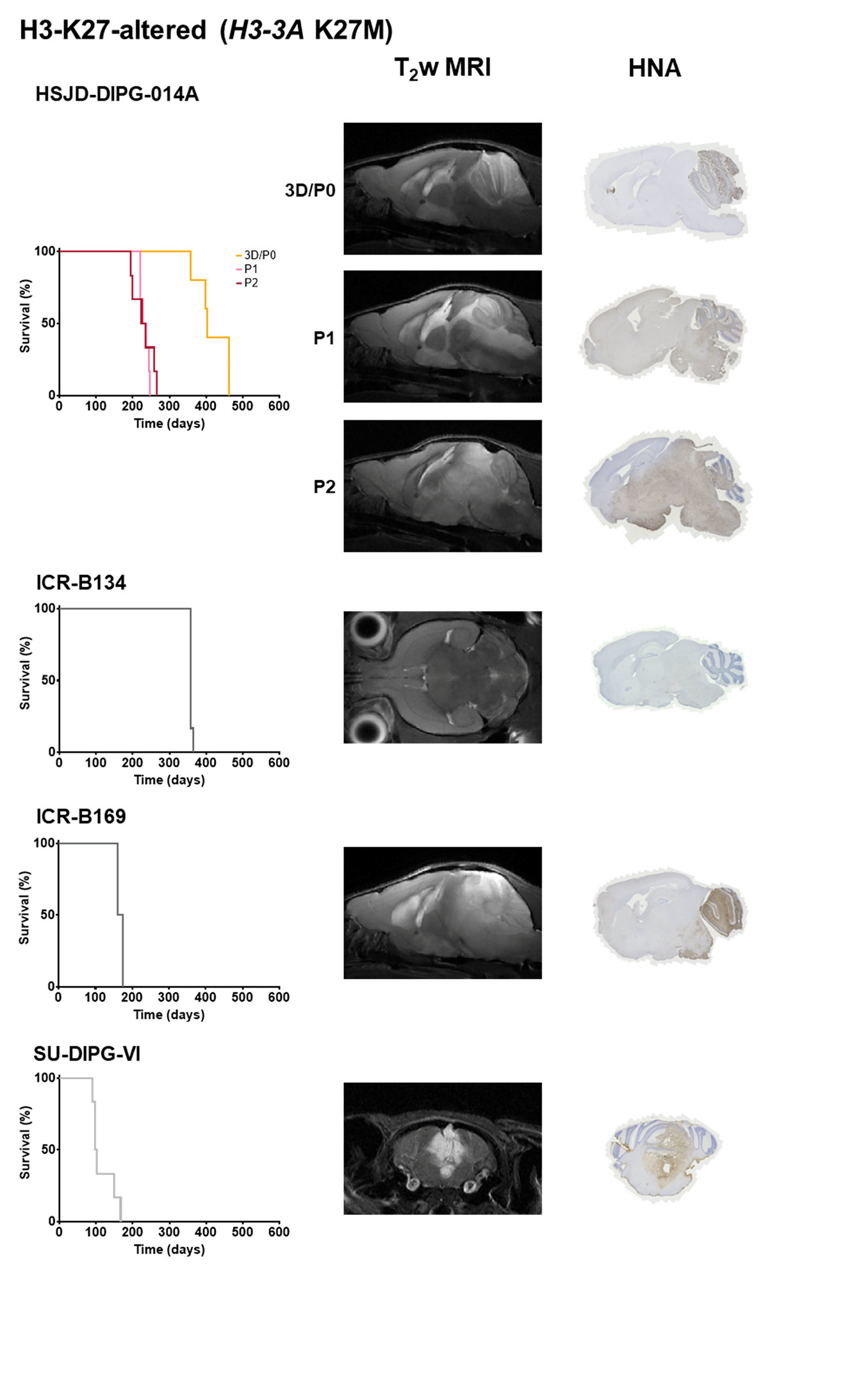
*

**Supplementary Figure S1**

*
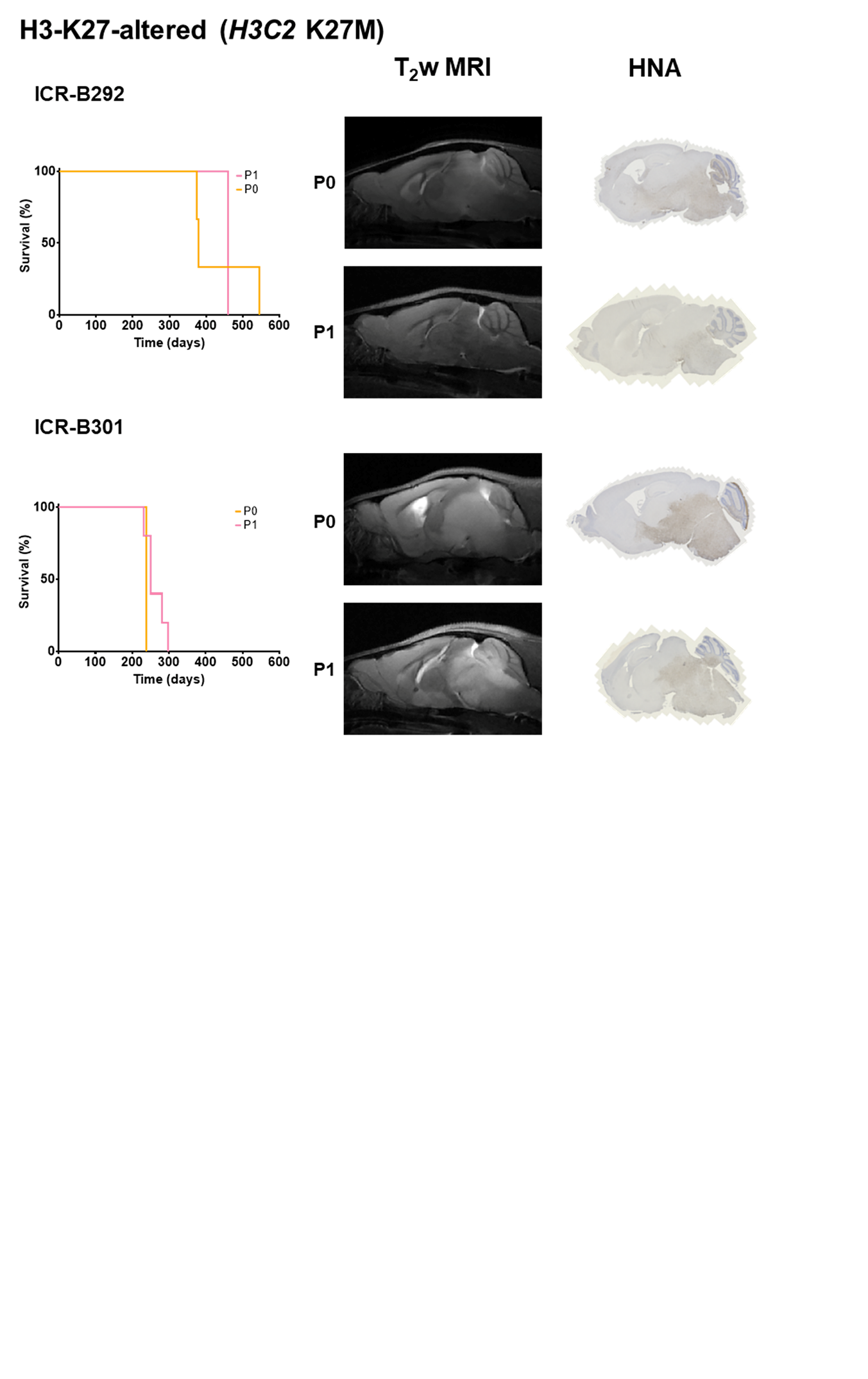
*

**Supplementary Figure S1**

*
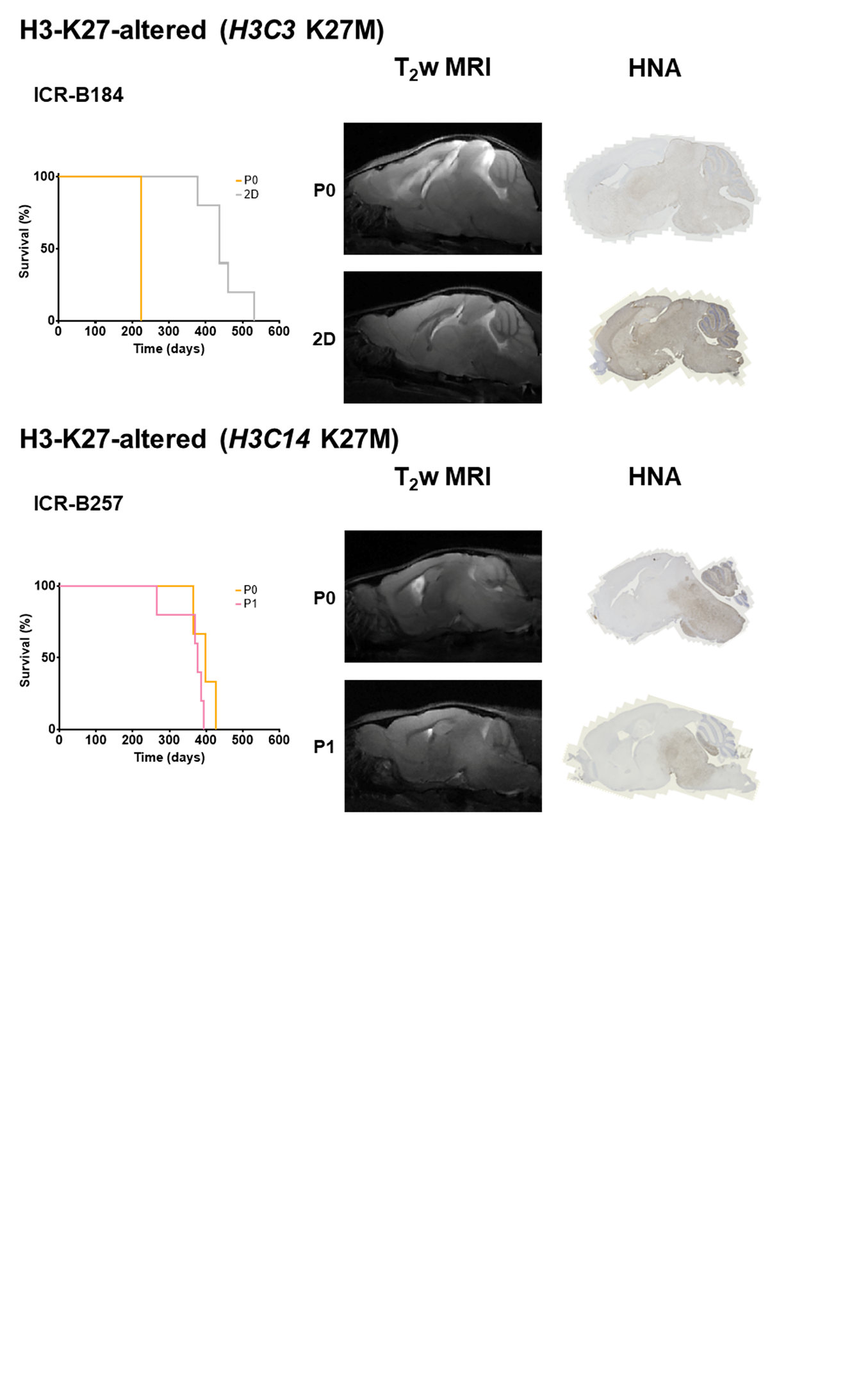
*

**Supplementary Figure S1**

*
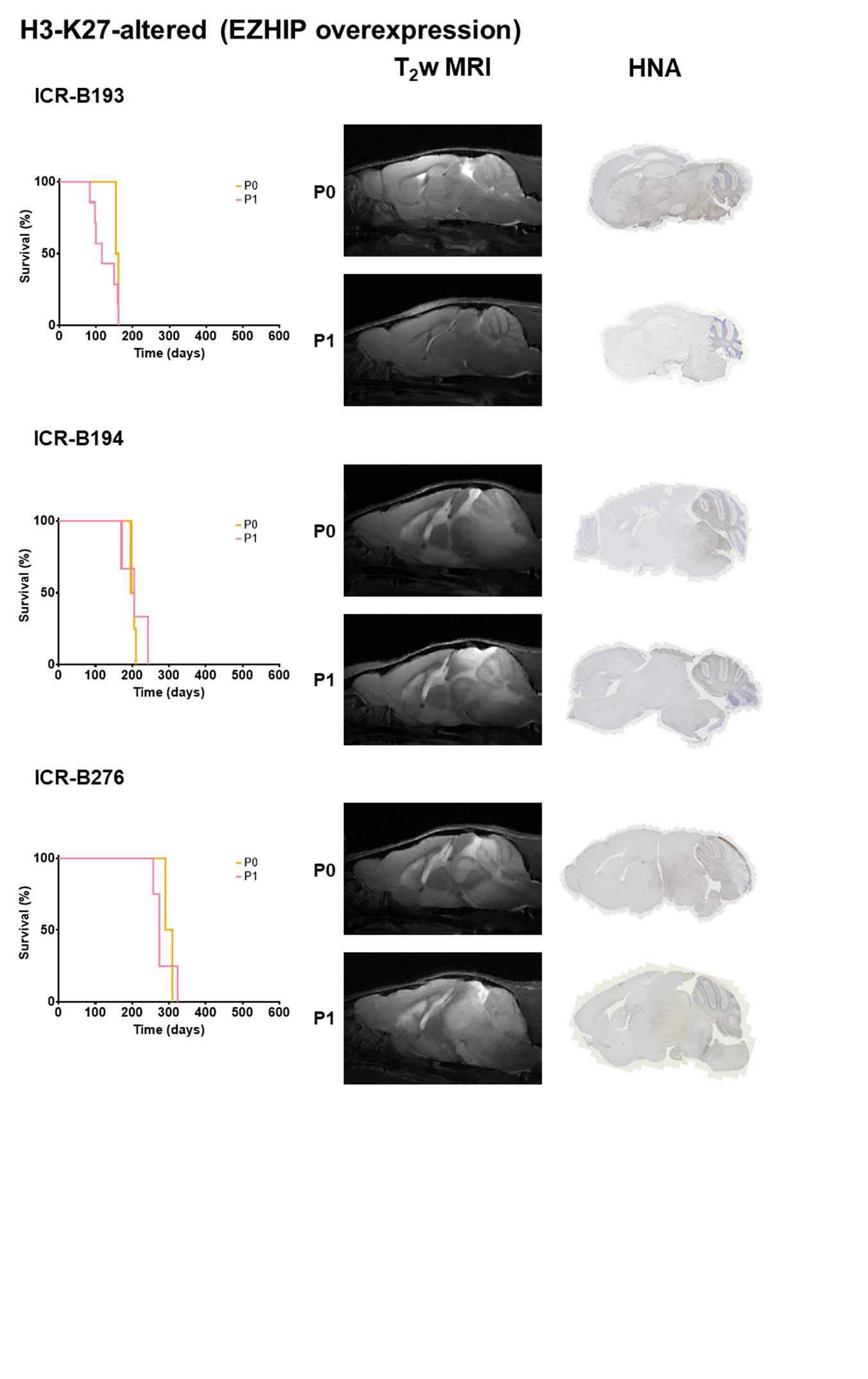
*

**Supplementary Figure S1**

*
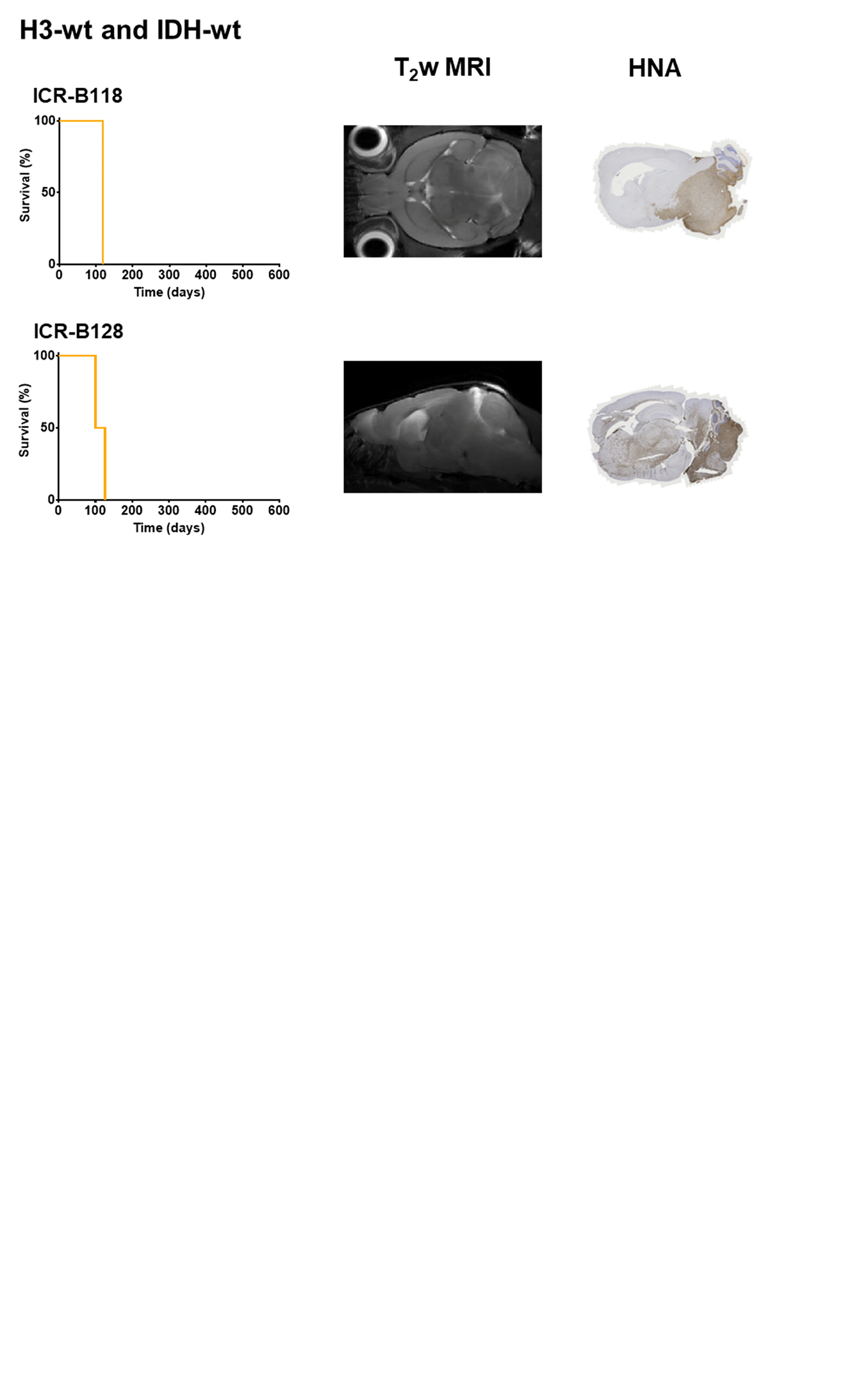
*

**Supplementary Figure S1: Summary survival, MRI and human nuclear antigen immunohistochemistry data for all patient-derived orthotopic *in vivo* models of PDHGG.**

Survival curves for all *in vivo* models of PDHGG alongside a T_2_-weighted (T_2_w) MR image and human nuclear antigen (HNA) staining of a representative tumour for each model, with slice position and orientation closely matched where possible. Models are separated by location and PDHGG subtype, including subtypes of brainstem DMG-H3-K27-altered tumours (*H3-3A/H3C2/H3C3/H3C14* K27M and EZHIP overexpression). 2D denotes mice injected with cells cultured in 2D on laminin (pale grey); 3D denotes mice injected with cells cultured in 3D as neurospheres (dark grey). P0 denotes original cohort of mice injected with cells directly from patient material (orange); 3D/P0 denotes original cohort of mice injected with cells cultured in 3D conditions as neurospheres (orange); P1 denotes mice injected with tumour cells isolated from a P0 mouse (pink); P2 mice were injected with tumour cells from a P1 mouse (red) cells; Where cells from multiple conditions or passages were implanted, images are labelled.

**Supplementary Figure S2**


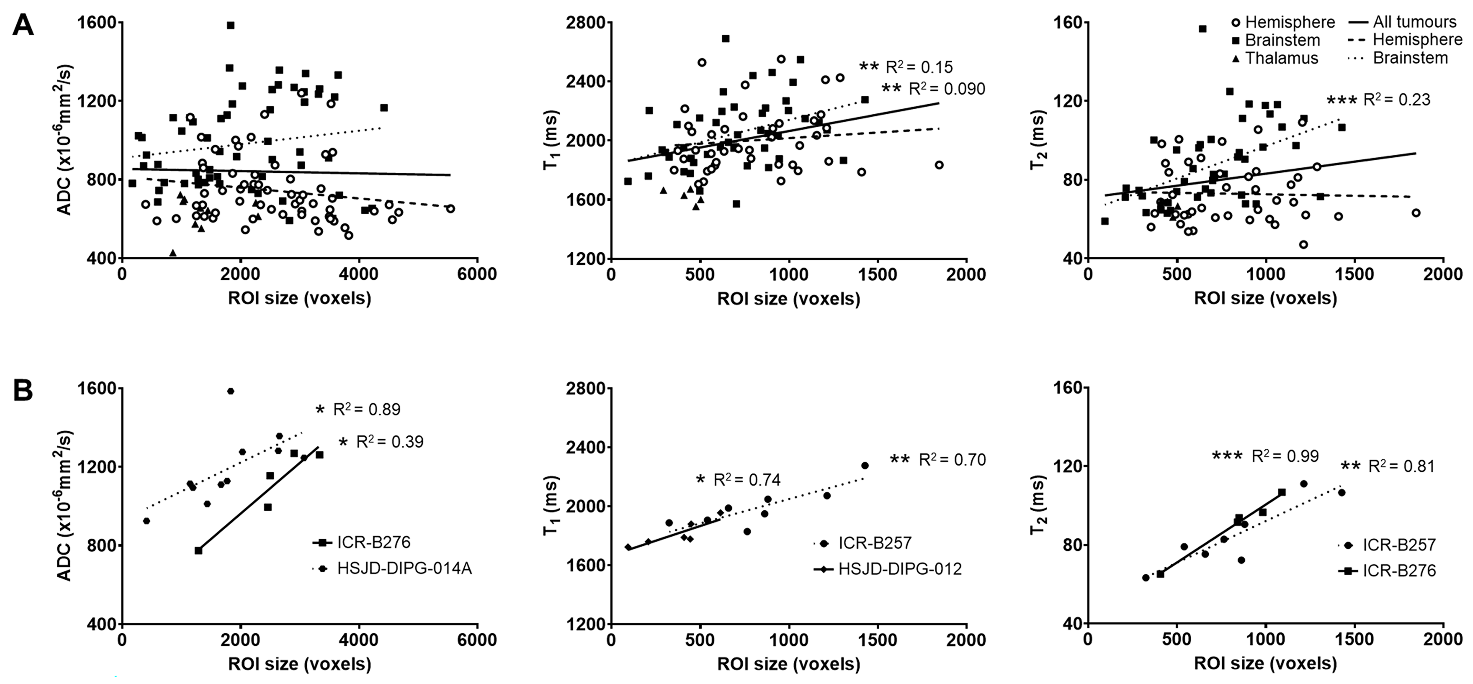


**Supplementary Figure S2: Relationship between region of interest (ROI) size and quantitative MRI parameters.**

**A.** Linear regression analysis of the relationship between ROI size and the MRI parameters apparent diffusion coefficient (ADC), T_1_ or T_2_ in all (solid line), hemispheric (dashed line) and brainstem (dotted line) tumours. DHG tumours are represented by open circles, thalamic DMGs by closed triangles and brainstem DMGs by closed squares. **B.** Linear regression analysis of the relationship between ROI size and ADC, T_1_ or T_2_ in individual tumour models in which a significant relationship was observed. * p < 0.05, ** p < 0.01, *** p < 0.001.

**Supplementary Figure S3**


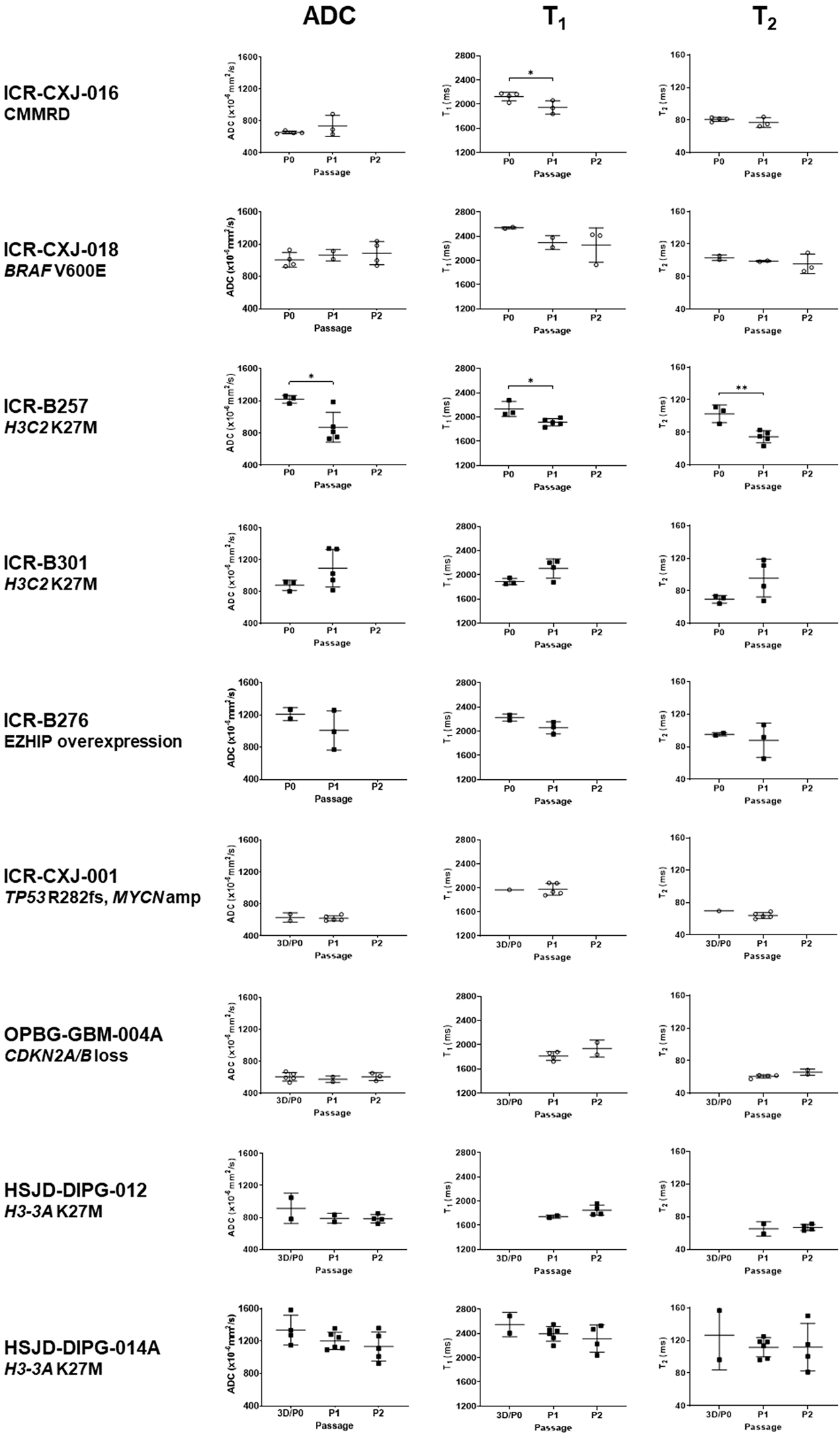


**Supplementary Figure S3: Quantitative MRI of serially xenografted PDHGG xenografts.**

Quantification of MRI parameters apparent diffusion coefficient (ADC), T_1_ and T_2_ from serially xenografted DHG-H3-wt (open circles) and brainstem DMG-K27-altered (closed squares) tumours. P0 denotes original cohort of mice injected with cells directly from patient material; 3D/P0 denotes original cohort of mice injected with cells cultured in 3D conditions as neurospheres; P1 denotes mice injected with tumour cells isolated from a P0 mouse; P2 mice were injected with tumour cells from a P1 mouse. CMMRD denotes constitutional mismatch repair deficiency. Data are mean ± S.D. of individual tumour median values (n=1-15 per model). Unpaired Student’s t-test, ** p < 0.01.

**Supplementary Figure S4**

**
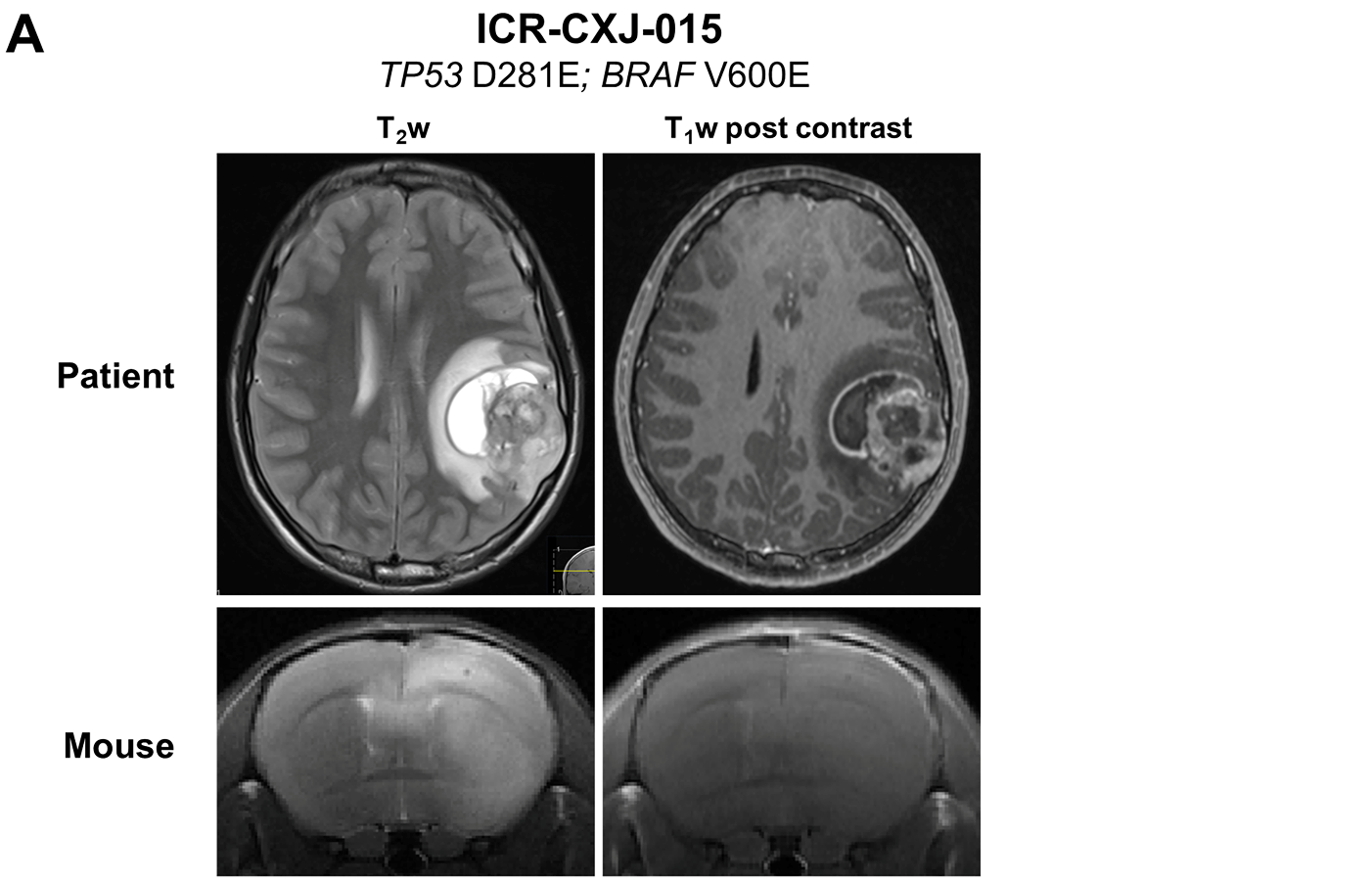

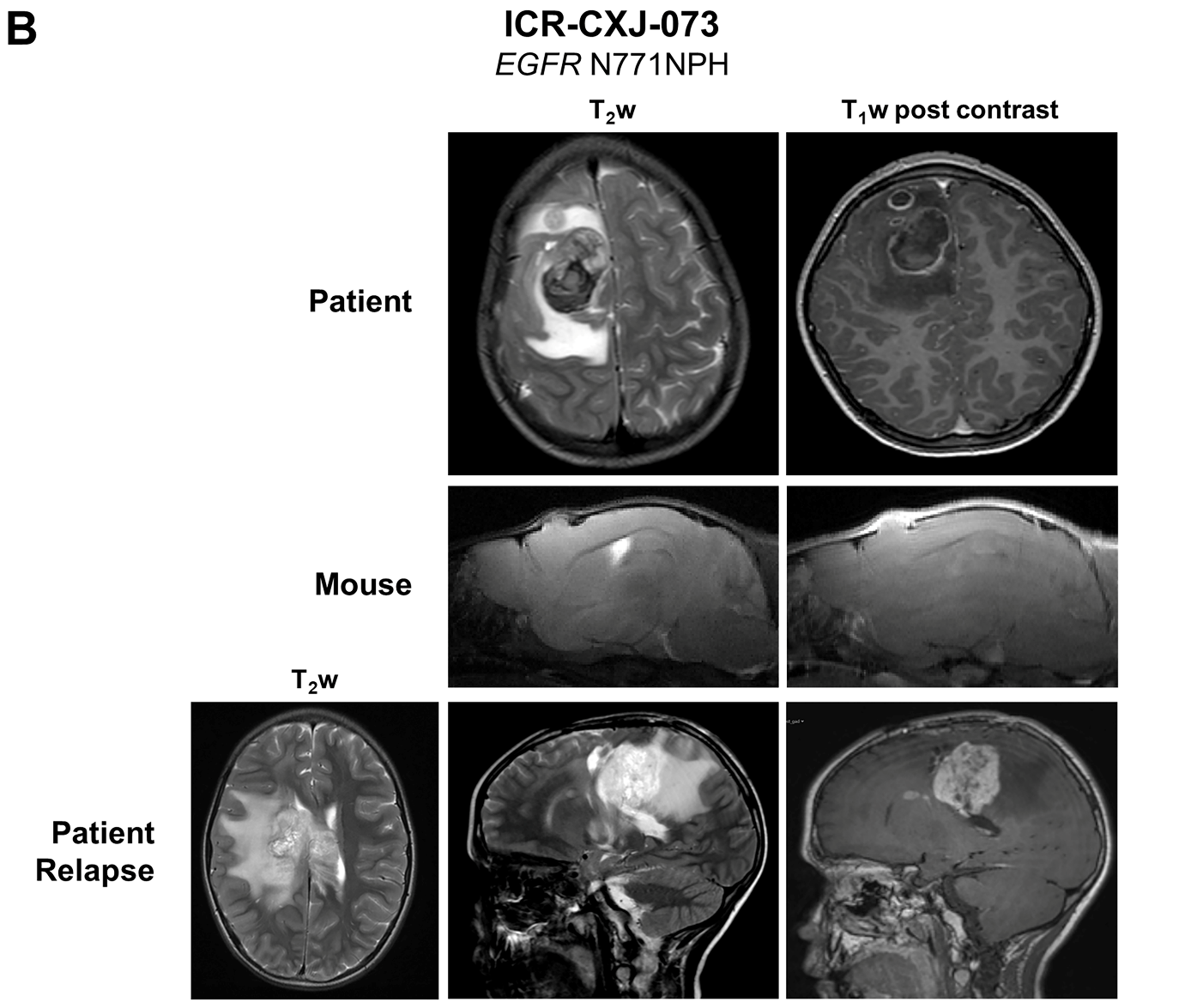
**

**Supplementary Figure S4**

**
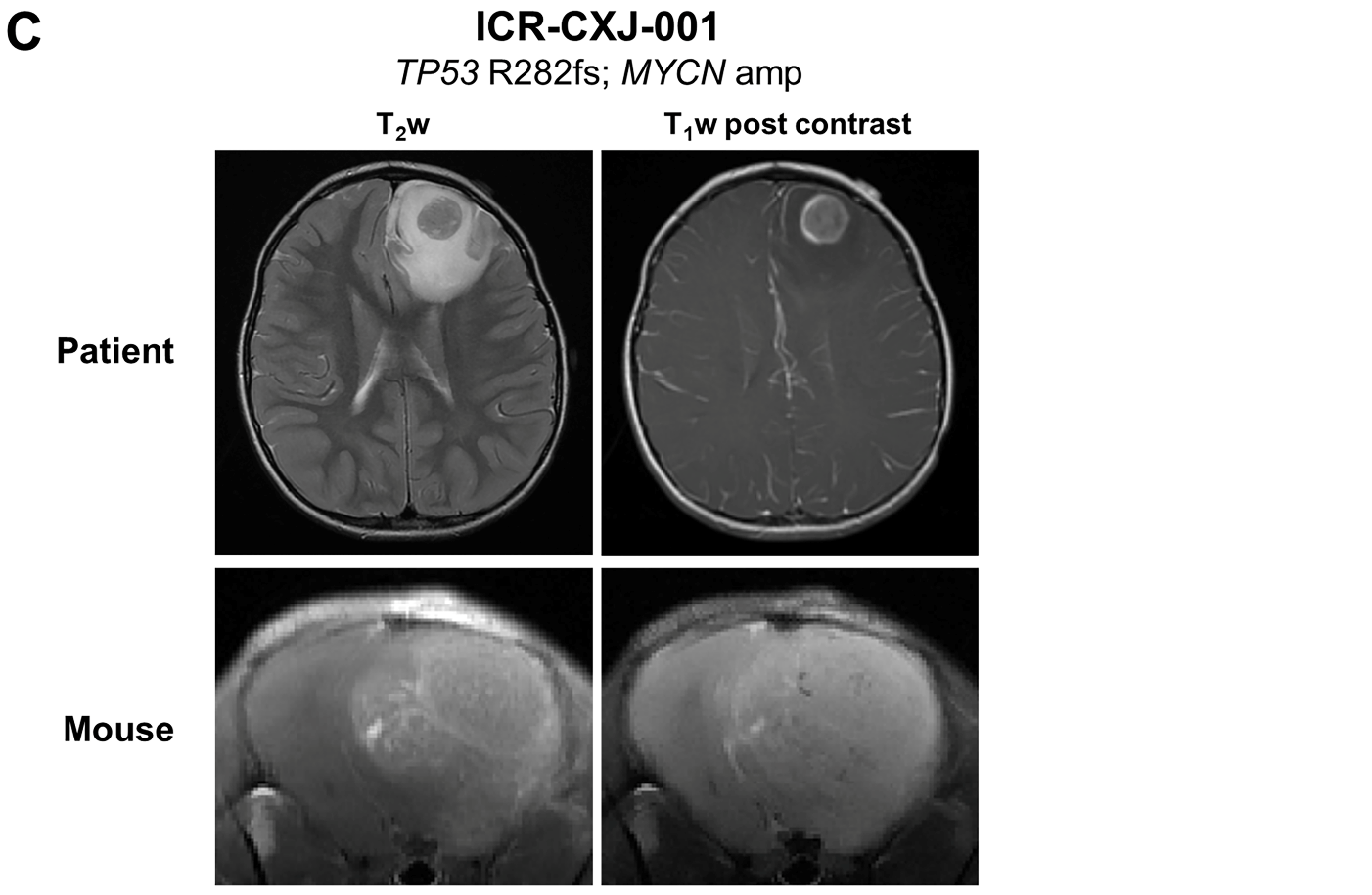

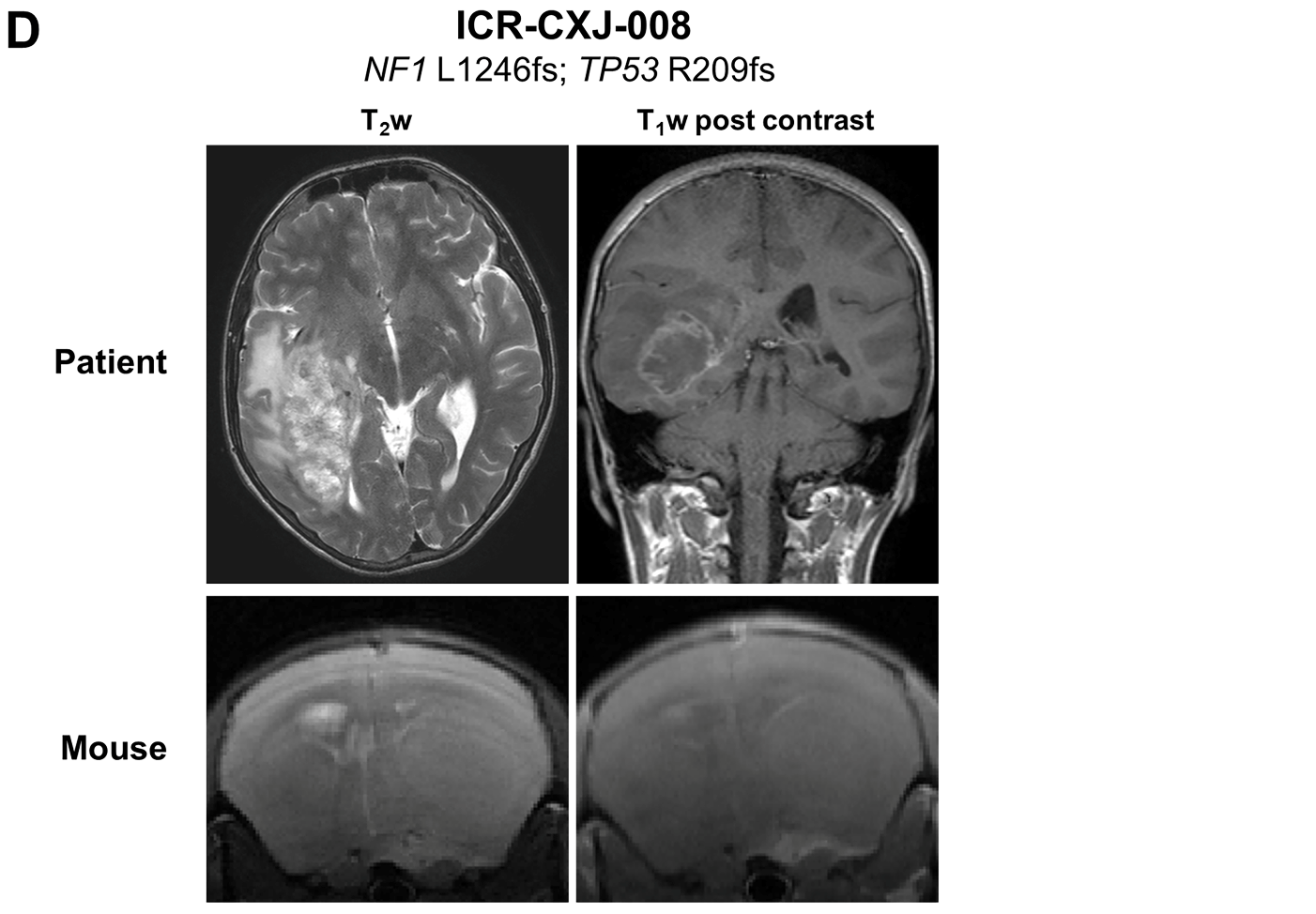
**

**Supplementary Figure S4**

**
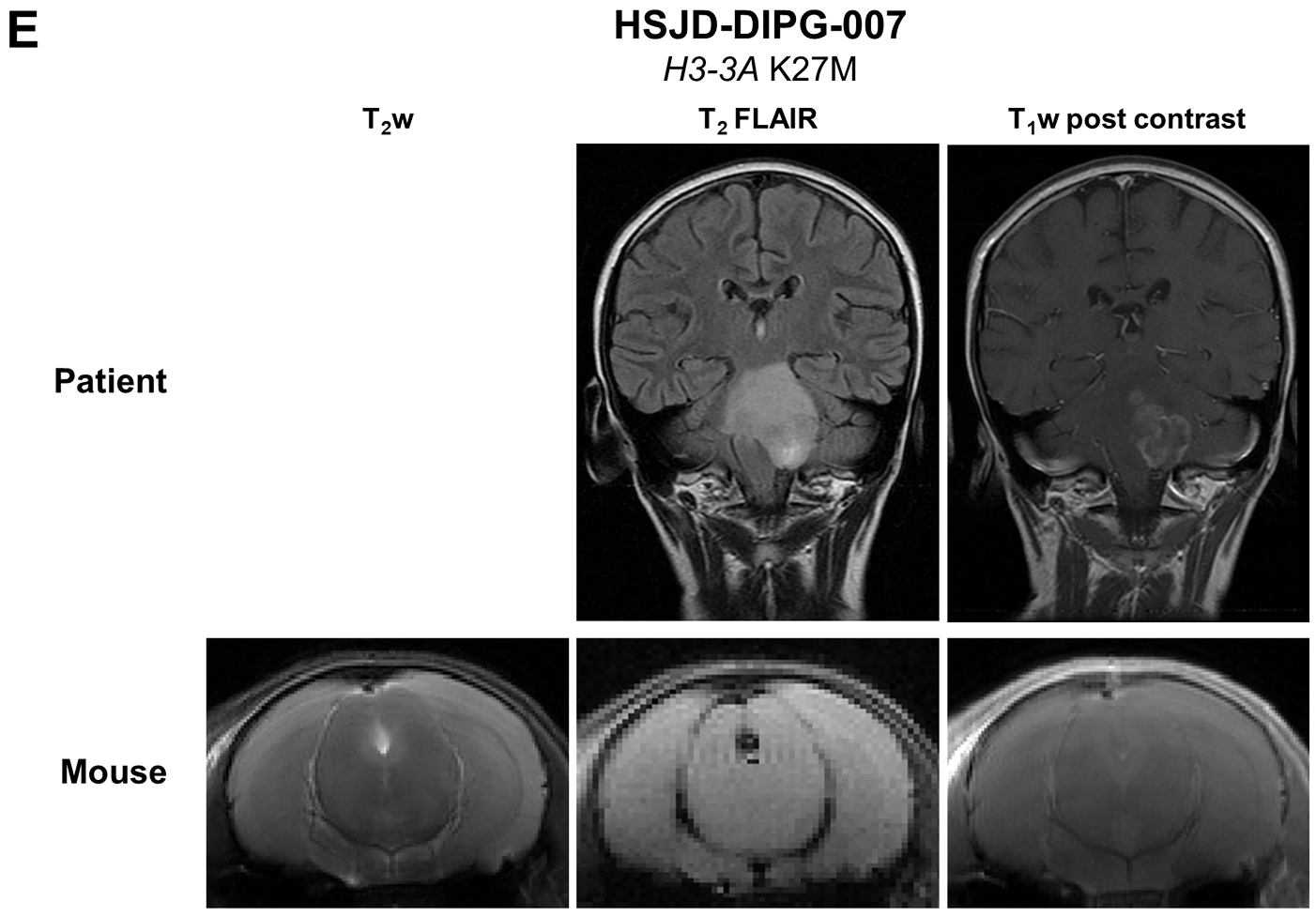

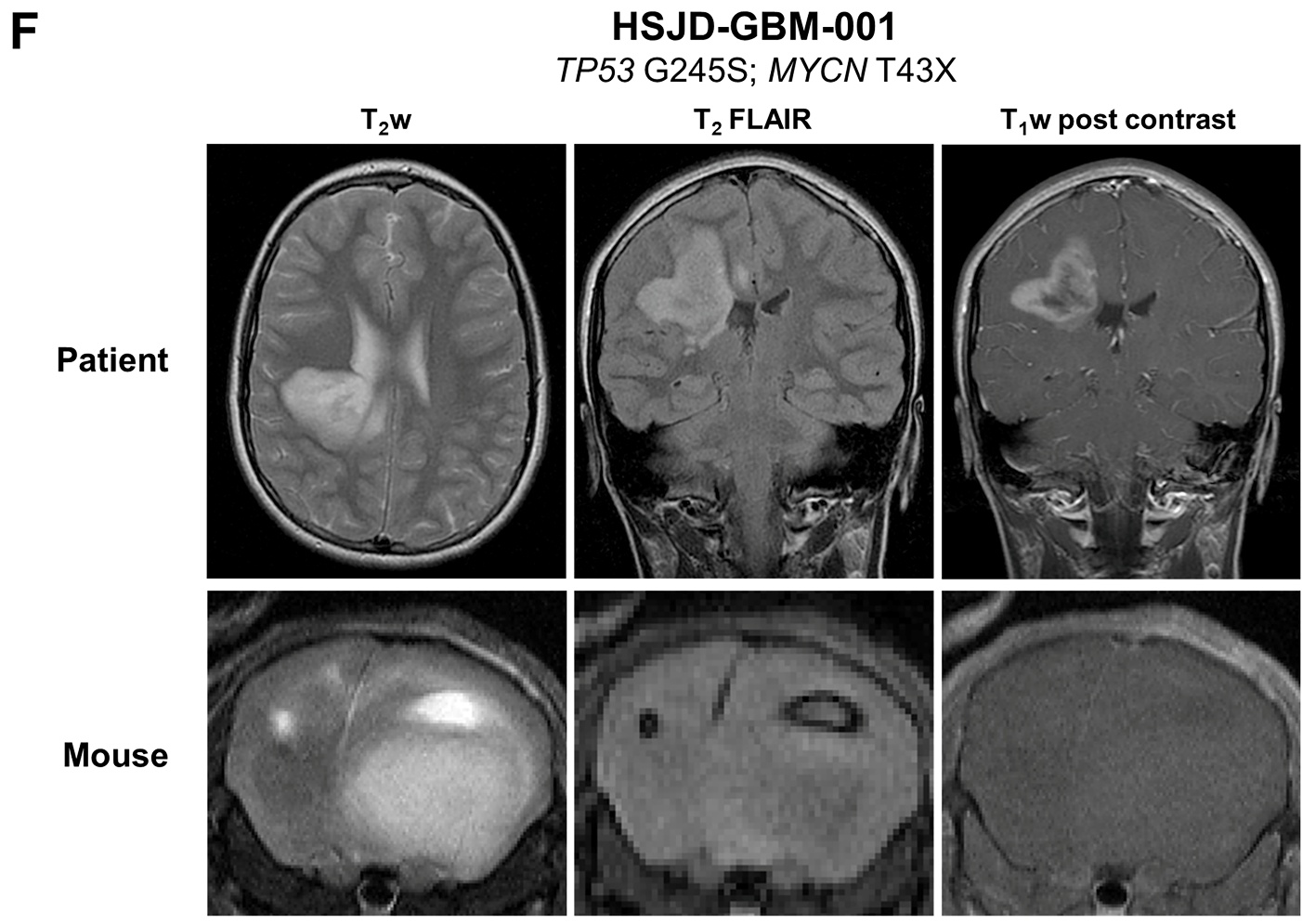
**

**Supplementary Figure S4**

**
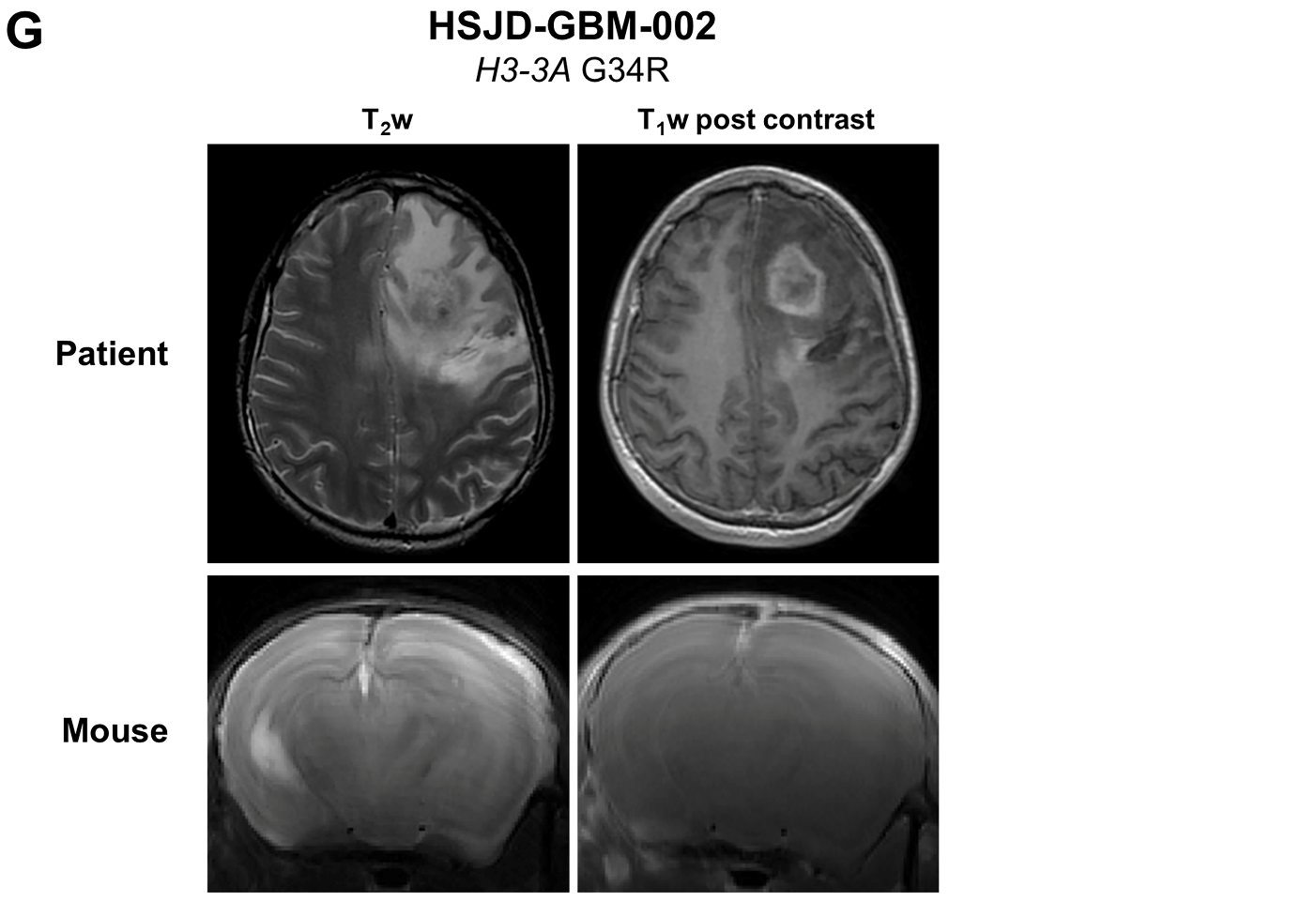

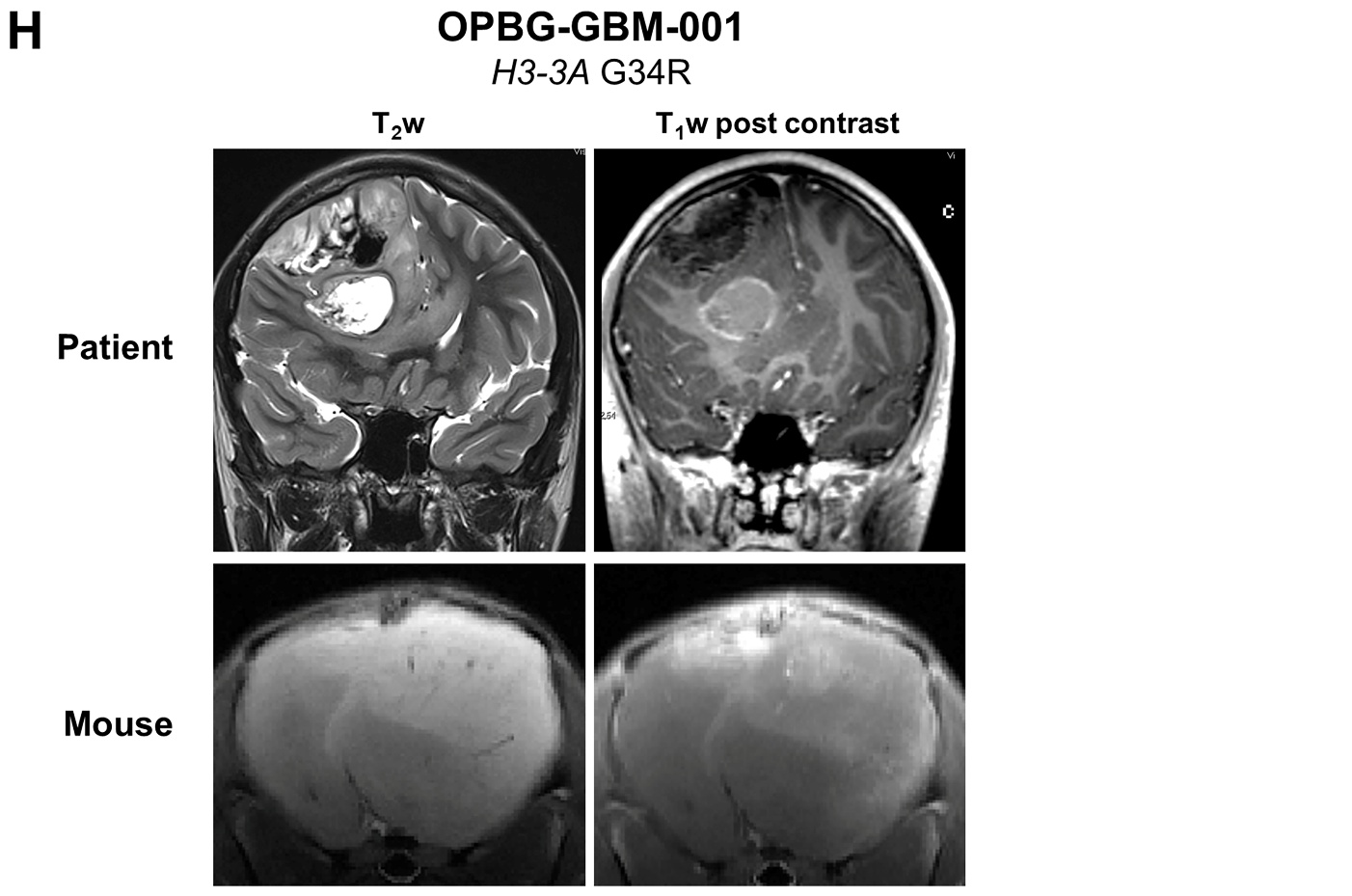
**

**Supplementary Figure S4**

**
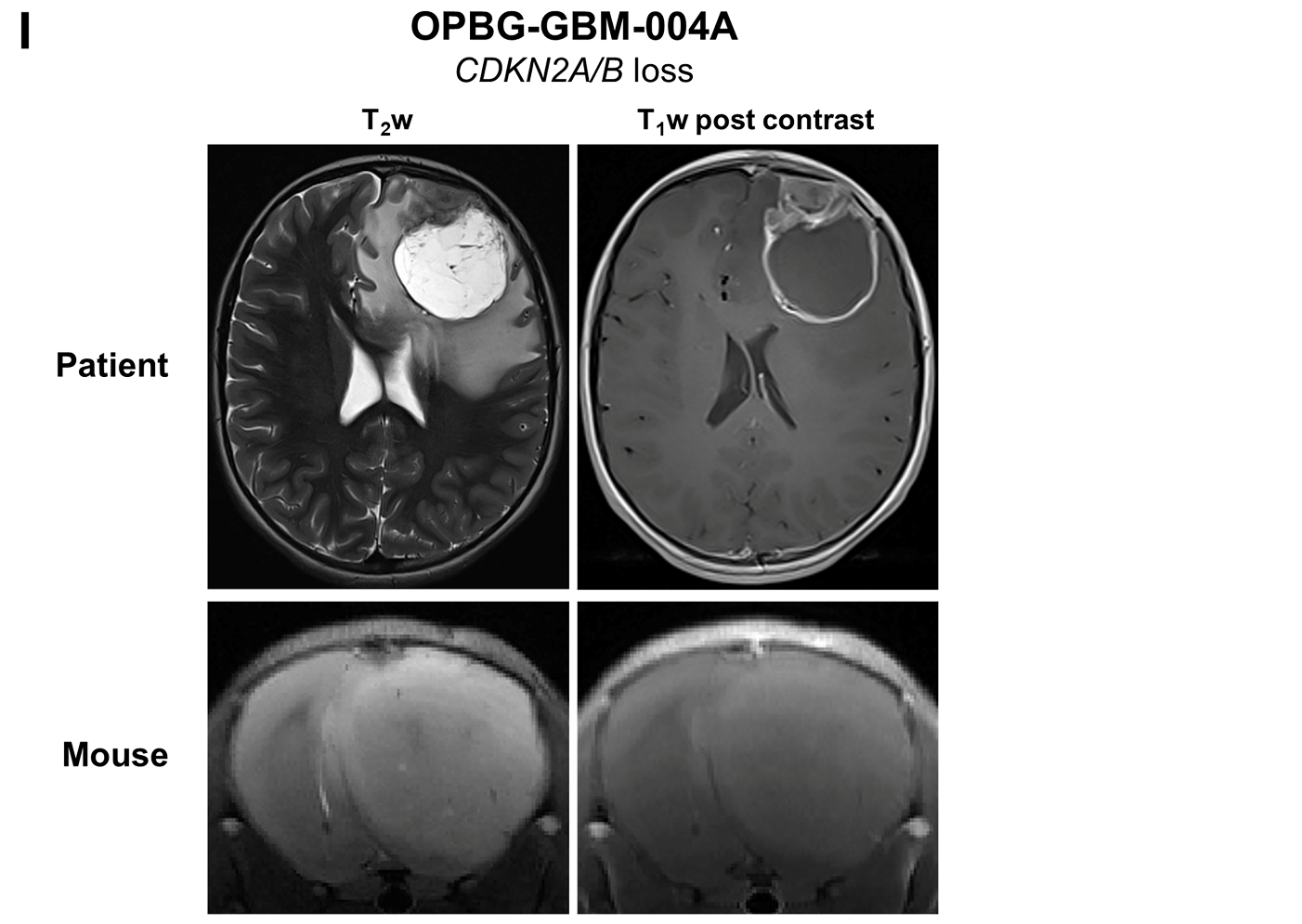
**

**Supplementary Figure S4: Magnetic resonance imaging from PDHGG patients and the corresponding *in vivo* mouse models.**

T_2_-weighted (T_2_w) and/or T_2_ fluid-attenuated inversion recovery (FLAIR) MR images alongside T_1_-weighted images acquired after intravenous administration of a gadolinium-based contrast agent (T_1_w post contrast) from PDHGG patients, close to the time of tissue collection where possible, and the corresponding *in vivo* mouse models. **A.** ICR-CXJ-015; Clinical imaging showed a heterogeneously enhancing part solid, part cystic, lesion with surrounding oedema. *In vivo* model displayed diffusely hyperintense lesion on T_2_w images and no contrast enhancement. **B.** ICR-CXJ-073; MRI at diagnosis showed a haemorrhagic tumour with associated oedema, limited contrast enhancement observed at border of haematoma. At relapse tumour had spread to contralateral hemisphere and displayed more extensive contrast enhancement. *In vivo* model displayed very diffuse growth with no BBB permeability resulting in diffuse hyperintensity on T_2_w images and no contrast enhancement. **C.** ICR-CXJ-001; Clinical imaging showed a tumour with well-defined T_2_ hypointense lesion with perilesional oedema and rim enhancement. *In vivo* model displayed focal oedema and heterogeneous contrast enhancement. **D.** ICR-CXJ-008; Clinical imaging showed a heterogeneous tumour and irregular peripheral enhancement. *In vivo* model displayed diffuse hyperintensity on T_2_w images and no contrast enhancement. **E.** HSJD-DIPG-007; Clinical imaging showed a hyperintense mass on T_2_ FLAIR with heterogeneous contrast enhancement. *In vivo* model displayed very diffuse hyperintensity on T_2_w images and no contrast enhancement. **F.** HSJD-GBM-001; Clinical imaging showed a T_2_ hyperintense tumour with a thick rim of peripheral enhancement. *In vivo* model displayed a well-defined T_2_ hyperintense tumour and no contrast enhancement. **G.** HSJD-GBM-002; Clinical imaging showed a heterogeneous T_2_ hyperintense tumour with peripheral enhancement. *In vivo* model displayed diffuse hyperintensity on T_2_w images and no contrast enhancement. **H.** OPBG-GBM-001; Clinical imaging showed an expansile solid-cystic lesion with diffuse infiltration and subtle heterogeneous contrast enhancement. *In vivo* model displayed a T_2_ hyperintense tumour with heterogeneous contrast enhancement. **I.** OPBG-GBM-004A; Clinical imaging of the relapsed tumour showed tumour tissue at the margins of the surgical cavity with necrosis, perilesional vasogenic oedema and contrast enhancement. *In vivo* model displayed a T_2_ hyperintense tumour with no contrast enhancement.
